# Academic Social Comparison and Social Mindfulness: The Roles of Self-Evaluation Threat and Regulatory Focus

**DOI:** 10.3390/bs16101856

**Published:** 2026-10-09

**Authors:** Qinhan Zhang, Litong Zhu, Weijian Li, Yuting Shao, Binghai Sun

**Affiliations:** 1 School of Psychology, Zhejiang Normal University, Jinhua 321004, China; qinhanzhang@zjnu.edu.cn (Q.Z.);; 2 Intelligent Laboratory of Child and Adolescent Mental Health and Crisis Intervention of Zhejiang Province, Zhejiang Normal University, Jinhua 321004, China; 3 School of Education, Zhejiang Normal University, Jinhua 321004, China

**Keywords:** social mindfulness, social comparison, self-evaluation threat, regulatory focus, hierarchical drift diffusion model

## Abstract

How does academic social comparison affect social mindfulness? Traditional perspectives largely attribute such changes to self-defensive unwillingness. Across three experiments involving academic-performance comparisons among university students in China, we examined social mindfulness using the Social Mindfulness (SoMi) paradigm and applied the Hierarchical Drift Diffusion Model (HDDM) to choices and response times in Experiments 1 and 2. Classical SoMi scores were lower following upward comparison than following downward or no comparison. Preference-adjusted scores showed the same directional pattern, although their post hoc pairwise differences did not remain significant after Holm correction. Experiment 2 further showed a significant indirect effect of upward comparison on social mindfulness through self-evaluation threat. HDDM analyses showed a drift-rate shift toward selecting the unique item following upward comparison. This model-based result indicates altered evidence accumulation between response options. Experiment 3 showed that promotion focus attenuated the differences in self-evaluation threat and social mindfulness. This behavioral finding is consistent with the possibility that motivational orientation shapes how upward comparison is interpreted. Together, these findings link upward academic comparison to reduced social mindfulness and changes in modelled decision processes, while suggesting that promotion focus may attenuate its behavioural consequences.

## 1. Introduction

Although the maxim “do not neglect to do good because it is small” is ancient, it underscores the fundamental nature of benevolent behavior. In contemporary social contexts, prosociality manifests through both high-cost charitable acts and daily micro-interactions. Social mindfulness (SoMi) concerns how people consider and preserve the options available to others when making their own choices (Van Doesum et al., 2013). For example, when choosing first from three blue pens and one black pen, selecting a blue pen leaves the next person a choice of colours, whereas selecting the only black pen removes that possibility. Either decision leaves the same number of pens available, but only the former preserves the other person’s opportunity to choose between alternatives. Such consideration typically entails little material sacrifice because the first chooser still receives an item, although foregoing a preferred option may carry a subjective cost.

Within the broader domain of prosocial behaviour, social mindfulness specifically concerns preserving others’ control over their subsequent choices. Whereas helping and charitable giving often involve providing assistance or transferring resources, socially mindful behaviour can benefit another person by maintaining their opportunity to act on their own preferences. Empathy and perspective taking may support this behaviour, but understanding another person’s situation does not necessarily lead to a decision that accommodates their interests. Social mindfulness combines an awareness of the consequences of one’s actions with a willingness to take those consequences into account (Van Doesum et al., 2013). Its interpersonal focus also distinguishes it from mindfulness as nonjudgmental awareness of present experience: social mindfulness concerns how such consideration is expressed through decisions that affect another person’s available options (Van Doesum et al., 2025).

Preserving others’ options involves recognising the interpersonal consequences of a choice and incorporating those consequences into the decision. In the SoMi paradigm, this means attending not only to which item one prefers but also to what selecting that item would leave for the next person. The social significance of the decision therefore depends on how individuals understand and evaluate the relationship between their own choices and another person’s opportunities. This makes social mindfulness relevant to situations in which attention to personal interests may compete with consideration for others.

Social comparison provides one such context. As a mechanism of self-evaluation, it shapes how individuals assess themselves in relation to others (Festinger, 1954). Comparing oneself with someone who performs better or worse may also influence subsequent interpersonal decisions. Previous research has linked the direction of comparison to differences in prosocial behaviour (Schlosser & Levy, 2016), but whether upward and downward comparison differentially affect the preservation of others’ choice opportunities remains unclear. The present study examines these effects in the context of academic-performance comparisons and investigates the role of self-evaluation threat (SET). By combining the SoMi paradigm with hierarchical drift diffusion modelling (HDDM), we further investigate how social comparison relates to the evidence accumulation underlying these decisions. Across three experiments, we examine the behavioural effects of comparison direction, the mediating role of self-evaluation threat, and whether a promotion focus buffers the effects of upward comparison.

### 1.1. The Influence of Social Comparison on Social Mindfulness

Current research on the influence of social comparison on prosocial behavior remains inconsistent, as this divergence stems from differing psychological mechanisms. Resource Conservation Theory posits that when individuals face upward comparators (i.e., find themselves in a position of relative disadvantage), they seek to restore self-integrity by conserving resources and reducing effort to compensate for this perceived status or psychological gap (Schlosser & Levy, 2016). Conversely, the social risk hypothesis suggests that low-status individuals strategically increase cooperation to align with high-status counterparts in response to resource uncertainty, thereby rendering prosocial behavior a utilitarian investment (Piff & Robinson, 2017). The activation of these mechanisms depends on situational factors, where the former emphasizes immediate self-protection and the latter is highly contingent upon the visibility of interaction partners and anticipated future benefits (Buunk & Gibbons, 2006).

The present study introduces the “asymmetry effect” of social comparison to elucidate these underlying mechanisms. In daily micro-interactions, the two directions of social comparison exert unequal influence. Although downward comparison may foster a more favourable self-evaluation, this psychological benefit need not translate into greater consideration of others’ choice opportunities. Instead, behavioral baselines are primarily disrupted by the sense of deprivation and inferiority induced by upward comparison, which activates self-preservation instincts (Gerber et al., 2018). Consequently, this study hypothesizes that the defensive motivation triggered by upward social comparison constitutes the core driver behind the lower social mindfulness (Van Doesum et al., 2025).

Building upon this analysis, we further delineate the theoretical boundaries of social mindfulness. Unlike high-visibility prosocial acts such as charitable giving, social mindfulness is characterized by its low cost and high cognitive involvement (Van Doesum et al., 2020). First, it typically manifests in anonymous or one-off micro-decisions, which precludes direct reciprocity and strategic calculations for cooperation. Second, although it does not require significant material sacrifice, it necessitates the expenditure of cognitive resources to monitor the choice spaces of others. These features raise the question of how upward comparison affects decisions that preserve others’ options. A reduction in social mindfulness could reflect changes in the attention given to another person’s options, the value assigned to preserving them, or both. Self-evaluation threat may contribute to these changes, but the resulting choice pattern alone cannot distinguish cognitive from motivational explanations. We therefore propose Hypothesis 1: upward social comparison will reduce social mindfulness relative to the no-comparison control condition, whereas downward comparison is not expected to produce a significant increase.

### 1.2. Self-Evaluation Threat: The Psychological Mechanism

Social comparison carries significant psychological implications that are far from neutral. Upward social comparison threatens the core self-concept by generating self-evaluation threats, which subsequently impair behavioral outcomes. According to the Self-Evaluation Maintenance (SEM) model (Tesser, 1988), when individuals perceive themselves as lagging behind others on critical dimensions, such as academic performance, they experience significant psychological deficits (Gerber et al., 2018; H. Liu et al., 2025). To restore psychological stability, individuals often activate defensive mechanisms (Aron, 2025; Zogmaister & Maricutoiu, 2022). In the context of social mindfulness, this defense manifests as a micro-level psychological compensation strategy, where individuals restrict the choices of others to regain an immediate sense of control or power (Schlosser & Levy, 2016).

A cognitive resource account offers one possible explanation alongside motivational accounts. The strength model of self-control proposes that regulating responses to psychological threat draws on resources that may also be needed for other activities (Baumeister et al., 1998). In the present context, concerns about one’s own academic standing may divert attention from the options available to another person. Research on the focusing effect of self-evaluation threat suggests that such concerns can narrow attention toward information relevant to one’s own evaluation (Muller & Butera, 2007). Applied to the SoMi task, this account predicts that the consequences of selecting the unique item may receive less attention during the decision. Alternatively, participants may recognise those consequences but place less value on preserving the other person’s options. Both processes could contribute to lower social mindfulness, and the present design cannot conclusively distinguish them.

These accounts suggest that self-evaluation threat may help explain the relationship between upward comparison and social mindfulness. Combining behavioural measures with computational modelling allows us to examine whether comparison-related differences in choice are accompanied by changes in evidence accumulation, without treating those changes as uniquely cognitive or motivational. We therefore propose Hypothesis 2: self-evaluation threat will mediate the relationship between upward social comparison and social mindfulness, such that upward comparison will be associated with greater threat, which in turn will be associated with lower social mindfulness.

### 1.3. Innovation in Cognitive Mechanisms: Evidence from the Hierarchical Drift Diffusion Model

Choice proportions indicate how often participants preserve another person’s options, but provide limited information about the decision process leading to those choices. Response times offer an additional source of information. The drift diffusion model (DDM) jointly models choices and response times by representing each decision as the accumulation of noisy evidence towards one of two response boundaries. A response occurs when the accumulated evidence reaches a boundary (Ratcliff et al., 2016).

The model separates several aspects of this process. Drift rate (*v*) describes the average direction and rate of evidence accumulation. Boundary separation (*a*) describes how much evidence is required before a response is made and is commonly interpreted as decision caution. Non-decision time (*t*) accounts for processes outside the decision stage, such as stimulus encoding and response execution. Starting-point bias (*z*) represents an initial tendency towards one boundary. In the SoMi task, the two response categories are selecting the unique item, which restricts the next person’s options, and selecting a non-unique item, which preserves those options. With the coding used in this study, a positive drift rate indicates accumulation towards the unique-item response.

The DDM describes the decision process on a trial. A hierarchical Bayesian DDM (HDDM) uses the same decision model but estimates participant-level parameters within group-level distributions. This allows information to be shared across participants while retaining individual differences, which is useful when each participant contributes relatively few experimental trials (Wiecki et al., 2013). We include comparison condition in this hierarchical model to examine differences in evidence accumulation, decision caution, and non-decision time. Threat associated with upward comparison has been linked to attentional focusing (Muller & Butera, 2007). We therefore expect upward comparison to shift evidence accumulation towards selecting the unique item. Such a shift would describe a change in the modelled decision process.

### 1.4. The Moderating Role of Regulatory Focus

Prior research suggests that self-evaluation threat and the subsequent depletion of cognitive resources may be the core mechanism by which upward comparison suppresses social mindfulness. We posit that the cognitive processing of comparative information is crucial for mitigating these threat perceptions. Regulatory Focus Theory (Higgins, 1997) provides a theoretical framework for this mechanism: individuals are driven by two distinct motivational systems that dictate how they interpret environmental comparative information. Prevention-focused individuals prioritize safety and responsibility, exhibiting heightened sensitivity to negative outcomes and potential losses (Molden et al., 2008; D. Liu et al., 2023). They are prone to encoding upward comparison gaps as potential threats to their status quo (Boissicat et al., 2022). Such an appraisal may heighten self-evaluation threat and increase attention to self-protective concerns, potentially reducing consideration of another person’s options during subsequent choices.

Conversely, promotion-focused individuals prioritize growth and ideals, focusing on opportunities for positive gains and self-improvement. Promotion focus may encourage individuals to interpret a superior comparison target as a potential role model or an opportunity for self-improvement (Lockwood et al., 2002). Such an interpretation could favour assimilation and reduce the self-evaluation threat associated with upward comparison.

At the cognitive level, an assimilative interpretation of a superior comparison target may reduce the self-evaluation threat associated with upward comparison. If less attention is drawn to self-defensive concerns, individuals may have greater scope to consider external social cues, including the options left for the subsequent chooser. Promotion focus may therefore support social mindfulness by shaping how upward comparison is interpreted and how the subsequent choice is approached. Accordingly, we propose that regulatory focus moderates the relationship between upward comparison, self-evaluation threat, and social mindfulness. Specifically, compared with a prevention focus, a promotion focus may attenuate upward-comparison-induced self-evaluation threat and thereby reduce the decline in social mindfulness.

### 1.5. Current Study

Across three experiments, we examined how academic social comparison affects social mindfulness and investigated the roles of self-evaluation threat and regulatory focus. Experiment 1 tested whether upward and downward comparison differentially affected social mindfulness and used HDDM to examine the associated decision processes. Experiment 2 assessed self-evaluation threat as a potential mediator of the comparison effect and examined its association with the estimated drift rate. Experiment 3 tested whether promotion focus, relative to prevention focus, attenuated the upward–downward differences in self-evaluation threat and social mindfulness. Together, these experiments combined behavioural measures, self-reports, and computational modelling to investigate the interpersonal consequences of academic social comparison.

## 2. Experiment 1

### 2.1. Method

#### 2.1.1. Participants

An a priori power analysis using G*Power 3.1 (*f* = 0.25, *α* = 0.05, power = 0.80) indicated a minimum sample size of 159 participants. We recruited 167 undergraduate students majoring in disciplines other than psychology from universities in China (*N_male_* = 85, *N_female_* = 82; *M_age_* = 20.86, *SD* = 1.18). Participants were randomly assigned to one of three conditions: the upward comparison group (*n* = 56), the downward comparison group (*n* = 56), or the no-comparison control group (*n* = 55).

#### 2.1.2. Materials

##### Trait Social Mindfulness

We assessed dispositional social mindfulness using Tian et al.’s (2021) 17-item Social Mindfulness Self-Report Scale (SMSRS), developed and validated in Chinese samples. The scale has four first-order factors: kindness and respect (four items, concerning consideration for and respect towards others), humility (five items, including yielding choice to others and avoiding self-promotion), tolerance and understanding (four items, concerning perspective taking and acceptance of differences), and positivity and openness (four items, concerning an optimistic and engaged orientation). In Tian et al.’s model, kindness and respect and humility form a higher-order factor termed agreeableness, whereas tolerance and understanding and positivity and openness form a higher-order factor termed extraversion.

The SMSRS differs from the commonly used Social Mindfulness (SoMi) choice paradigm in both scope and measurement. The SMSRS assesses a broader self-reported interpersonal disposition, whereas the SoMi paradigm measures whether a person’s choice in a particular situation preserves options for the next chooser (Van Doesum et al., 2013). In the present study, we used the SMSRS total mean score as a baseline measure of dispositional social mindfulness; we did not test or analyse its four factors separately. Items were rated on a five-point scale, with higher scores indicating greater dispositional social mindfulness. Internal consistency for the total score was Cronbach’s α = 0.74.

##### Social Comparison Manipulation and Manipulation Check

Following the protocol established by Zheng et al. (2015), we induced social comparison using real-world academic performance. Participants were instructed to compare their academic performance from the previous academic year with that of a classmate from the same major and grade who ranked either first (upward comparison condition) or last (downward comparison condition). To reinforce the manipulation, participants indicated their approximate relative standing on a percentile rank scale ranging from 1% to 100%. We included a single manipulation check item stating, “I believe my academic performance is better than that of this classmate,” which was rated on a 5-point Likert scale from “strongly disagree” to “strongly agree.” Additionally, we assessed the personal subjective importance of academic performance to ensure the relevance of the comparison domain.

##### State Social Mindfulness Assessment

**Figure 1:**
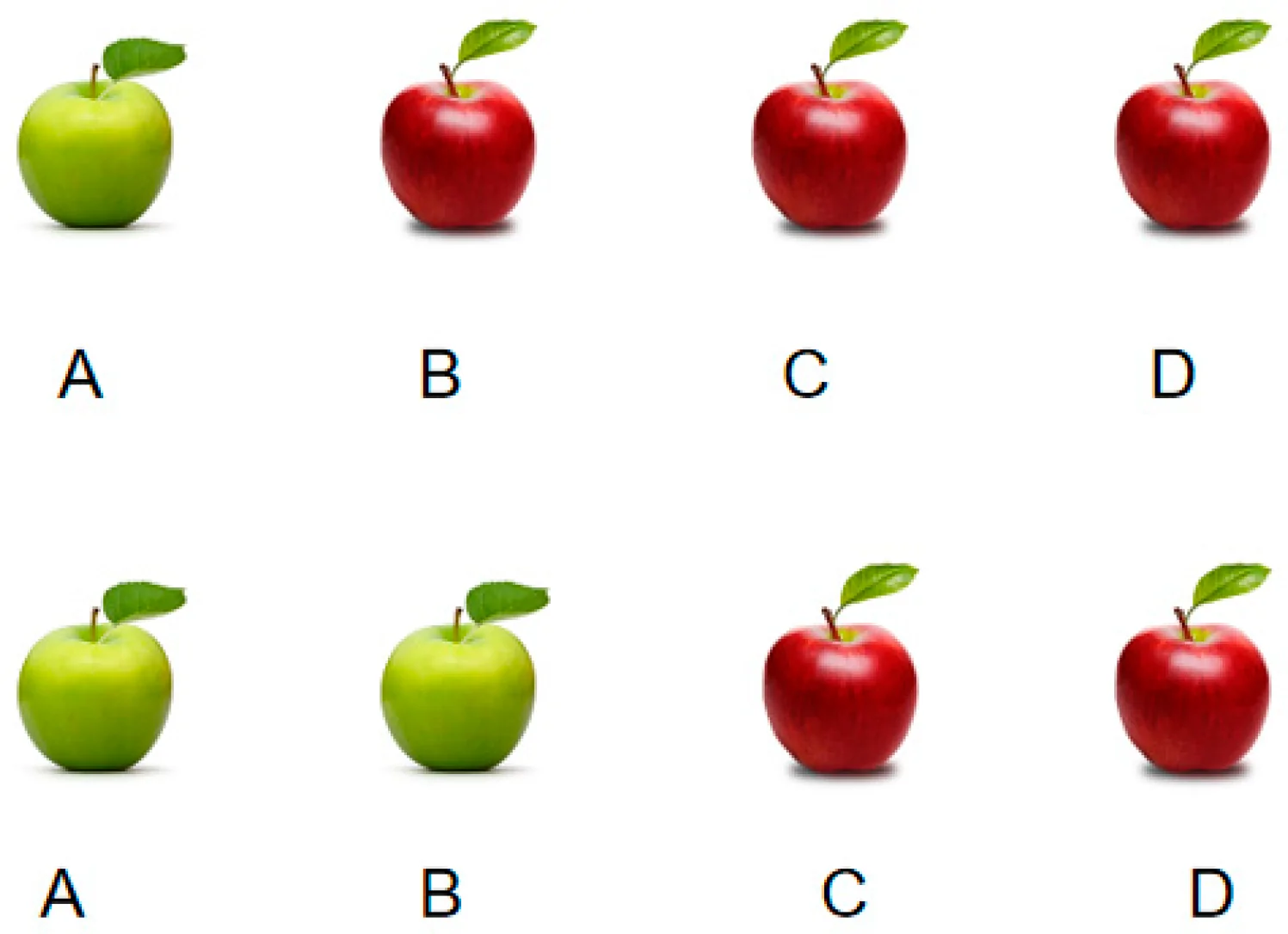
Example of an experimental trial in the SoMi paradigm.

We assessed state social mindfulness using the SoMi paradigm (Van Doesum et al., 2013). All participants completed 24 object-selection trials: 12 experimental trials and 12 matched control trials, presented in a randomized order (see Figure 1). Each of 12 object categories appeared once in each trial type. On every trial, participants were told that they would choose first from four objects and that an anonymous partner would choose one of the remaining objects.

In an experimental trial, three objects were of one type and one was of another type (a 3:1 arrangement). Selecting the unique object left the partner with three objects of the same type, whereas selecting one of the three non-unique objects left the partner a choice between the two types. A non-unique selection was therefore scored as socially mindful. In the corresponding control trial, two objects of each type were available (a 2:2 arrangement). Whichever type the participant selected, both types remained available to the partner. These control trials were used to assess the participant’s choice between the two object types when neither choice removed a type from the partner’s options.

The classical SoMi score was the proportion of the 12 experimental trials on which a participant selected a non-unique object. For the preference-adjusted SoMi score (SoMi_Adj), an experimental non-unique choice was counted when, in the matched control trial, the participant had selected the object type that was unique in the experimental trial. This score thus identifies non-unique choices made despite an indicated preference for the unique object type. Control trials did not contribute directly to the classical SoMi score or to the HDDM analysis.

#### 2.1.3. Data Analysis Strategy

We fitted a hierarchical Bayesian DDM to choices and response times from the 12 experimental SoMi trials. Selecting the unique item, which restricted the second chooser’s options, was coded as the upper boundary; selecting a non-unique item, which preserved those options, was coded as the lower boundary. A positive drift rate therefore indicated evidence accumulation towards the unique-item response. After applying the response-time criteria, the analysis included 1486 trials from 167 participants. Detailed trial exclusions and response coding are reported in the Supplementary Material S6.

Drift rate (*v*), boundary separation (*a*), and non-decision time (*t*) were allowed to vary by comparison condition; starting-point bias (*z*) was estimated with participant-level intercepts but was not allowed to vary by condition. We fitted the model in brms. Condition differences were evaluated using direct posterior contrasts calculated from the retained posterior draws. For each contrast, we report the posterior mean, the 95% equal-tailed credible interval (CrI), and the posterior probability of the difference in the reported direction. Contrasts for boundary separation and non-decision time were calculated on the model’s log scale. Parameter traces and posterior predictive response-time checks are shown in Supplementary Figures S1 and S2. The Supplementary Material S6 provide the distributional specification, priors, software versions, and sampling settings. As a sensitivity analysis, we refitted the HDDM for Experiments 1 and 2 using a 4.0-s rather than a 3.0-s upper response-time cutoff. The 0.3-s lower cutoff, model specification, and priors were unchanged; results are reported in Supplementary Material S6.

For each SoMi outcome in Experiments 1 and 2, we used a one-way ANOVA to test the overall difference among the three comparison conditions. We then conducted two-sided independent-samples *t* tests for all three pairwise comparisons. These comparisons were treated as post hoc, and their *p* values were adjusted using the Holm method separately within each experiment and outcome. Holm-adjusted *p* values were used for interpretation; unadjusted *p* values are additionally reported for the primary preference-adjusted comparisons.

For the preference-adjusted SoMi outcome, we examined residual distributions and variance patterns in the ANOVA and linear outcome-regression models. Given the bounded and discrete nature of this measure, we additionally fitted participant-level quasi-binomial regression models with a logit link, using the number of trial pairs meeting the preference-adjustment criterion and the remaining pairs out of 12. The models included social comparison condition in Experiments 1 and 2 and its interaction with regulatory focus in Experiment 3. Outcome-regression sensitivity analyses additionally included self-evaluation threat as a predictor and examined HC3 standard errors for the corresponding linear models. Diagnostic and robustness results are reported in Supplementary Material S9.

#### 2.1.4. Experimental Procedure

Participants first provided demographic information and completed the assessment for trait social mindfulness. Subsequently, participants were randomly assigned to one of three experimental conditions to complete the corresponding social comparison task, which involved reviewing grades and marking their rank, followed by manipulation check items. The control group was exempt from these specific steps. Finally, all participants completed the SoMi paradigm task consisting of 24 object-selection trials. Instructions were reiterated before each trial to maintain the salience of the social interaction context. The 24 SoMi trials were presented in a randomized order, and the spatial arrangement of objects within each trial was also randomized.

### 2.2. Results

#### 2.2.1. Extraneous Variables and Manipulation Check

Preliminary analyses confirmed no significant differences in objective academic ranking, subjective grade importance, or trait social mindfulness across groups (all *p* > 0.05; see Supplementary Material S1 for details). The manipulation was effective: participants in the downward comparison condition perceived their performance as significantly better (*M_down_* = 4.59) than those in the upward comparison group (*M_up_* = 1.54; *t*(110) = −16.66, *p* < 0.001).

#### 2.2.2. Behavioral Results

**Figure 2:**
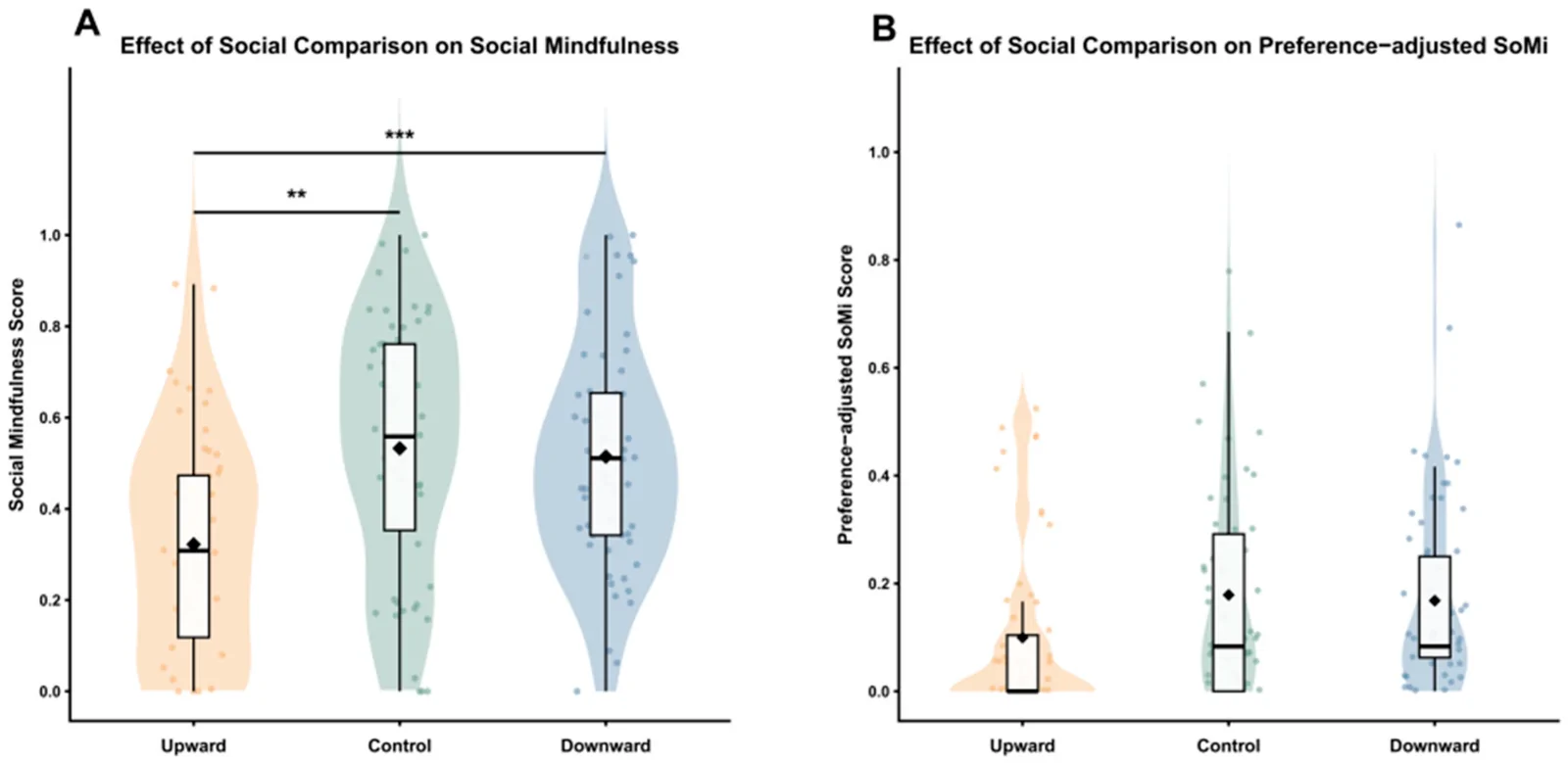
Social mindfulness and preference-adjusted social mindfulness scores across social comparison conditions. (**A**) Classical social mindfulness scores. (**B**) Preference-adjusted social mindfulness scores. Error bars denote 95% confidence intervals (CIs). Scatter points represent individual data. Brackets indicate Holm-adjusted pairwise comparisons. *** *p* < 0.001, ** *p* < 0.01.

We prioritized the preference-adjusted SoMi score to account for personal sacrifice (see Figure 2B). A one-way analysis of variance (ANOVA) yielded a significant main effect of condition (*F*(2, 164) = 3.32, *p* = 0.039, *η_p_^2^* = 0.039). The upward comparison group had a lower mean preference-adjusted SoMi score (*M* = 0.10) than the downward (*M* = 0.17; *t*(110) = −2.16, unadjusted *p* = 0.033, Holm-adjusted *p* = 0.066, Cohen’s *d* = 0.41) and control groups (*M* = 0.18; *t*(109) = −2.39, unadjusted *p* = 0.019, Holm-adjusted *p* = 0.056, Cohen’s *d* = 0.45). Neither pairwise difference remained statistically significant after Holm correction. Classical SoMi scores also showed a significant overall condition effect, and both comparisons involving the upward group remained significant after Holm correction (see Figure 2A and Supplementary Material S2). A quasi-binomial robustness analysis also supported the overall comparison-condition effect, *F*(2, 164) = 3.50, *p* = 0.032 (see Supplementary Material S9).

#### 2.2.3. Cognitive Mechanisms: Evidence from HDDM

Parameter traces and posterior predictive response-time checks are shown in Supplementary Figures S1 and S2. The predictive checks compare observed and model-predicted response-time distributions overall and by comparison condition.

**Figure 3:**
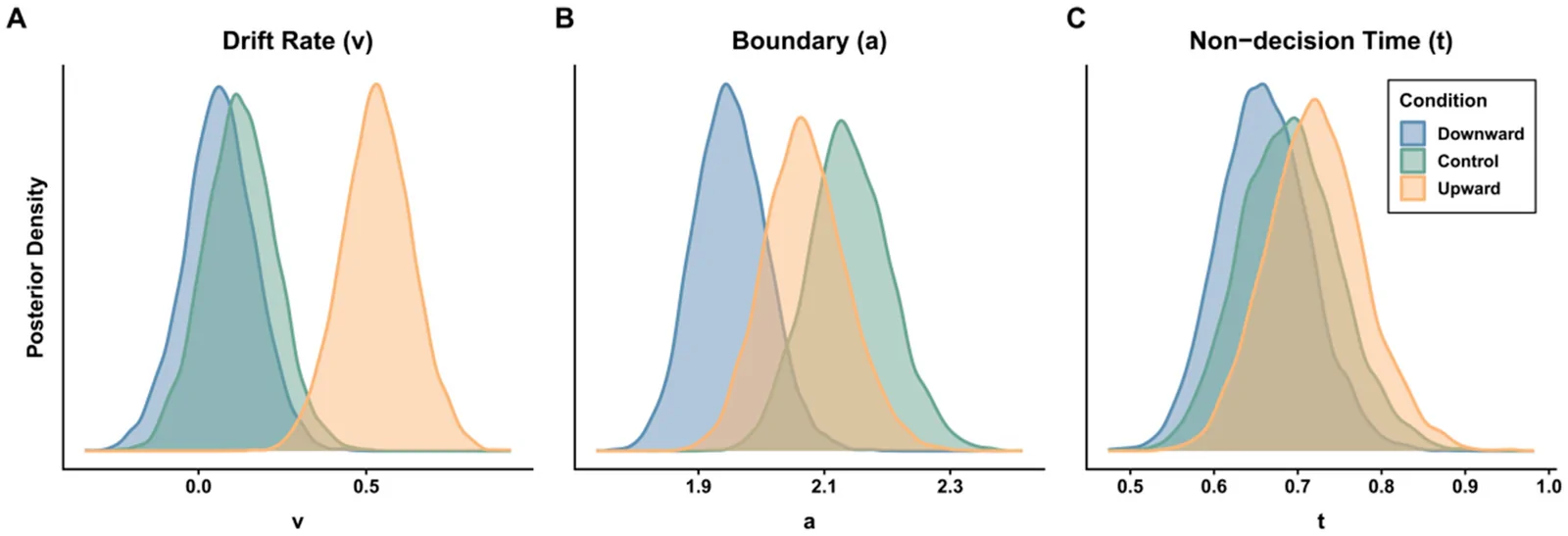
Posterior density distributions of HDDM parameters across conditions. Note: The figure displays the posterior distributions for DDM parameters under Downward (blue), Control (green), and Upward (orange) conditions. (**A**) Drift Rate (*v*), (**B**) Boundary Separation (*a*), (**C**) Non-decision Time (*t*). The separation between the density curves indicates the magnitude of the difference between conditions.

Bayesian parameter estimation revealed distinct cognitive profiles across social comparison conditions (see Figure 3). Direct posterior contrasts indicated a higher drift rate under upward comparison than under downward comparison (Δ*v* = 0.470, 95% CrI [0.193, 0.751], P(Δ*v* > 0) = 0.999) and the control condition (Δ*v* = 0.410, 95% CrI [0.132, 0.695], P(Δ*v* > 0) = 0.997). The downward–control contrast was less conclusive (Δ*v* = −0.061, 95% CrI [−0.350, 0.223]). For boundary separation, the upward–downward and upward–control contrast intervals included zero. However, boundary separation was lower under downward comparison than under the control condition on the model’s log scale (Δlog(a) = −0.093, 95% CrI [−0.174, −0.011], P(Δlog(a) < 0) = 0.986). All pairwise contrasts for non-decision time had 95% CrIs that included zero. Full pairwise posterior contrasts for drift rate, boundary separation, and non-decision time are reported in Supplementary Material S6, Table S1.

### 2.3. Summary

Experiment 1 showed lower classical SoMi scores following upward comparison than following downward or no comparison; preference-adjusted scores showed the same direction, but the pairwise differences were not statistically significant after Holm correction. This behavioural difference was accompanied by a drift-rate shift toward selecting the unique item. However, Experiment 1 did not measure self-evaluation threat and therefore could not establish whether this experience was associated with the observed differences. Experiment 2 addressed this question by examining self-evaluation threat as a potential mediator of the comparison effect on social mindfulness and assessing its association with the estimated drift rate.

## 3. Experiment 2

### 3.1. Method

#### 3.1.1. Participants

An a priori power analysis using G*Power (*f* = 0.25, *α* = 0.05, power = 0.80) indicated a minimum sample size of 159. We recruited 164 undergraduate students majoring in disciplines other than psychology from universities in China (*N_male_* = 75, *N_female_* = 89; *M_age_* = 21.04, *SD* = 1.57). Participants were randomly assigned to the upward comparison group (*n* = 55), the downward comparison group (*n* = 54), or the no-comparison control group (*n* = 55).

#### 3.1.2. Materials

##### Trait Social Mindfulness

The assessment protocol was identical to Experiment 1. The Cronbach’s *α* coefficient for the scale was 0.80.

##### Social Comparison Manipulation and Manipulation Check

To manipulate social comparison, we adopted the virtual character profile paradigm from Mussweiler (2001), specifically adapting the academic comparison materials from Chen (2018). Participants in the upward comparison group read a profile of a fictional student with superior academic performance. Conversely, participants in the downward comparison group read a profile of a fictional student with inferior academic performance. Following the reading task, participants responded to a single attention check item: “Based on the information provided, whose academic ranking is better, yours or this student’s?” Data from participants who answered incorrectly were excluded. The manipulation check consisted of three items, including “I consider my learning ability to be stronger than this student,” “I consider myself more excellent than this student,” and “I consider myself more likely to succeed than this student.” These items were rated on a 5-point Likert scale ranging from “strongly disagree” to “strongly agree.”

##### Measurement of Self-Evaluation Threat

Drawing on the use of a brief self-value evaluation measure in Wang et al. (2024), we assessed current self-evaluation immediately after the social comparison manipulation using three items: “I feel good about myself,” “I am confident in my abilities,” and “I can effectively handle any unexpected events.” Participants responded on a seven-point scale. All three items were reverse-scored and averaged, with higher scores indicating less positive current self-evaluation, which was used as an indirect indicator of self-evaluation threat. Internal consistency in Experiment 2 was satisfactory (Cronbach’s α = 0.87).

We used this composite as an indirect indicator of self-evaluation threat. According to the self-evaluation maintenance model, comparison with a better-performing other in a self-relevant domain can threaten positive self-evaluation (Tesser, 1988). In the present academic comparison context, the three items captured participants’ current self-regard, confidence in their abilities, and perceived coping capability. Administering them immediately after the comparison manipulation situated these evaluations within the comparison context. Lower ratings were therefore interpreted as a self-evaluative response consistent with threat.

##### State Social Mindfulness Assessment

We employed the SoMi paradigm, utilizing the identical protocol as in Experiment 1.

#### 3.1.3. Experimental Procedure

The experimental procedure consisted of four phases. First, trait social mindfulness was measured for all participants to establish a baseline level. Subsequently, participants were randomly assigned to their respective groups. The upward and downward comparison groups completed the virtual character comparison task and the corresponding manipulation checks, while the control group was exempt from this task. Third, participants were required to immediately complete the self-evaluation threat scale to capture their instantaneous psychological state. Finally, all participants completed the SoMi paradigm task. The trial-level choice and response-time data were analysed using the same hierarchical DDM specification and response coding as in Experiment 1. After applying the trial-level exclusion criteria, the HDDM analysis included 1323 experimental trials from 163 participants (54 in the upward-comparison group, 54 in the downward-comparison group, and 55 in the control group). One participant in the upward-comparison group had no eligible trials and therefore no individual drift-rate estimate, but remained in the behavioral analyses.

Mediation analyses were conducted in R 4.6.0 using the lavaan package (version 0.6-21). Social comparison condition was represented by two indicator variables for upward and downward comparison, with the no-comparison condition as the reference group. Self-evaluation threat was the mediator; preference-adjusted and classical SoMi scores were examined as separate outcomes. Indirect effects were evaluated using 5000 nonparametric bootstrap samples and 95% percentile confidence intervals. No covariates were included.

### 3.2. Results

#### 3.2.1. Extraneous Variables and Manipulation Check

Baseline analyses revealed no significant differences in trait social mindfulness among the three groups (*F*(2, 161) = 1.45, *p* = 0.238). The manipulation was successful: the upward comparison group perceived their academic ranking as significantly lower (*M* = 1.87) compared to the downward comparison group (*M* = 4.19; *t*(107) = −21.46, *p* < 0.001).

#### 3.2.2. The Impact of Social Comparison Direction on Social Mindfulness

**Figure 4:**
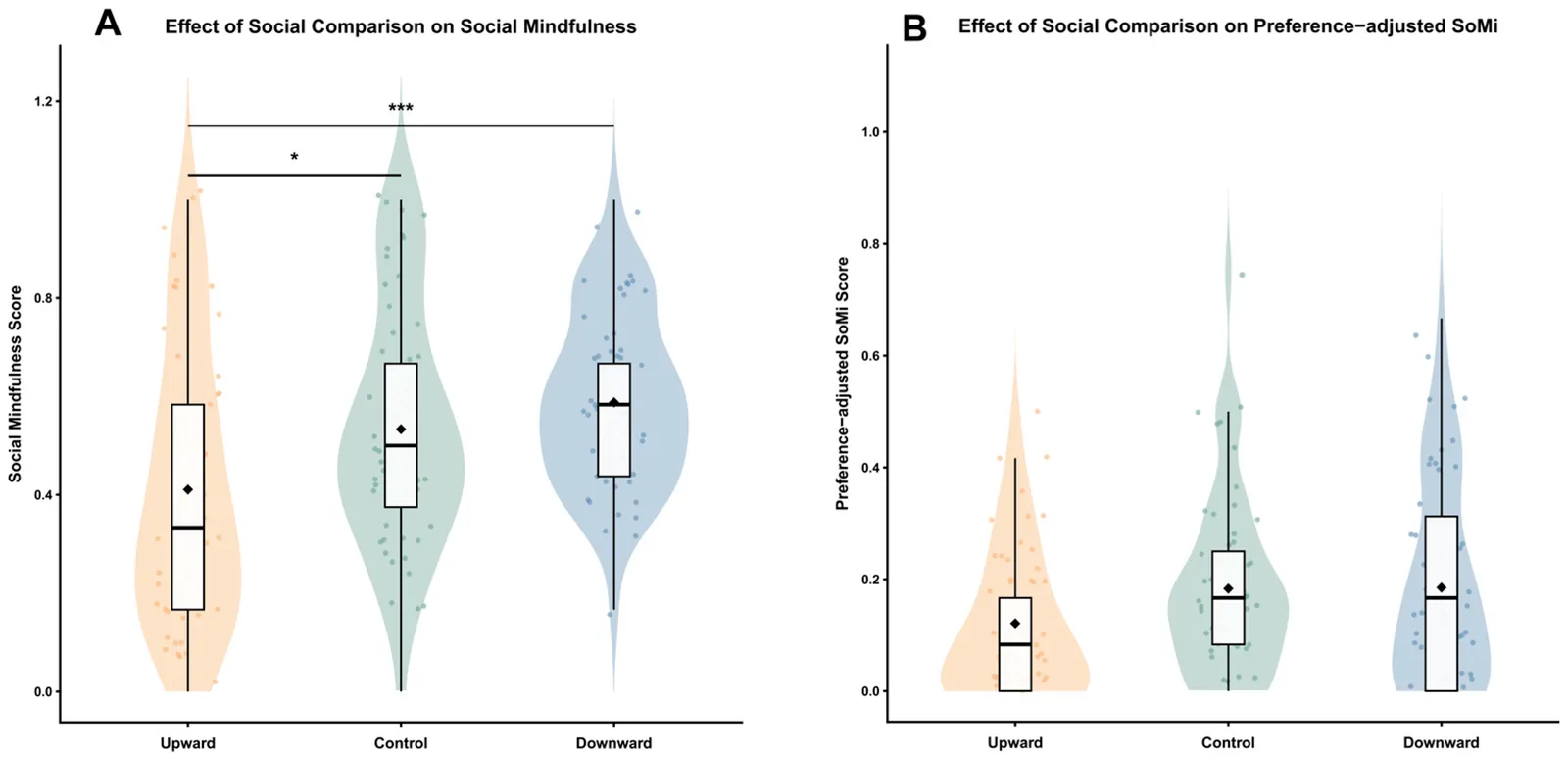
Social mindfulness and preference-adjusted social mindfulness scores across social comparison conditions in Experiment 2. (**A**) Classical social mindfulness scores. (**B**) Preference-adjusted social mindfulness scores. Error bars denote 95% confidence intervals (CIs). Scatter points represent individual data. Brackets indicate Holm-adjusted pairwise comparisons. *** *p* < 0.001, * *p* < 0.05.

The preference-adjusted SoMi scores showed the same directional pattern as in Experiment 1, but the overall condition effect did not reach statistical significance, *F*(2, 161) = 2.87, *p* = 0.060, η*_p_*^2^ = 0.034 (see Figure 4B). The upward comparison group had a lower mean score (M = 0.12) than the downward (*M* = 0.19; *t*(107) = −2.10, unadjusted *p* = 0.038, Holm-adjusted *p* = 0.084, Cohen’s *d* = 0.40) and control groups (*M* = 0.18; *t*(108) = −2.23, unadjusted *p* = 0.028, Holm-adjusted *p* = 0.084, Cohen’s *d* = 0.42). Neither pairwise comparison remained significant after Holm correction. Classical SoMi scores showed a significant overall condition effect, *F*(2, 161) = 8.10, *p* < 0.001, η*_p_*^2^ = 0.091 (see Figure 4A and Supplementary Material S2). The quasi-binomial omnibus test for preference-adjusted scores did not reach statistical significance, *F*(2, 161) = 2.77, *p* = 0.066 (see Supplementary Material S9).

#### 3.2.3. The Mediating Role of Self-Evaluation Threat

**Figure 5:**
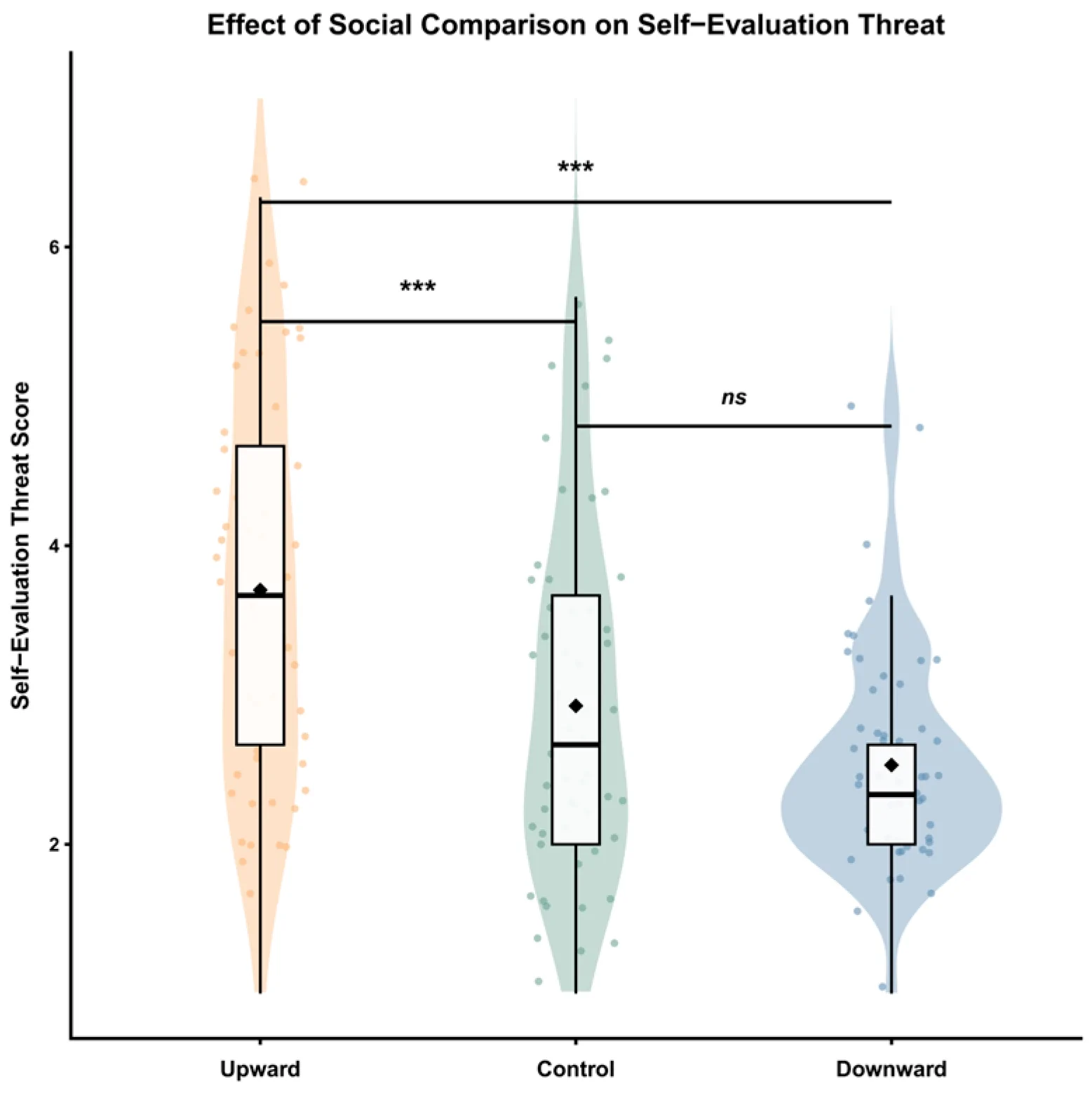
Self-evaluation threat scores across social comparison conditions in Experiment 2. *** *p* < 0.001; ns, not significant.

Regarding the psychological mechanism, as shown in Figure 5, upward comparison induced significantly higher SET (*M* = 3.71) compared to the downward (*M* = 2.53; *t*(107) = 5.53, *p* < 0.001, Cohen’s *d* = 1.06) and control groups (*M* = 2.93; *t*(108) = 3.67, *p* < 0.001, Cohen’s *d* = 0.70) (*F*(2, 161) = 15.860, *p* < 0.001, *η_p_*^2^ = 0.17).

**Figure 6:**
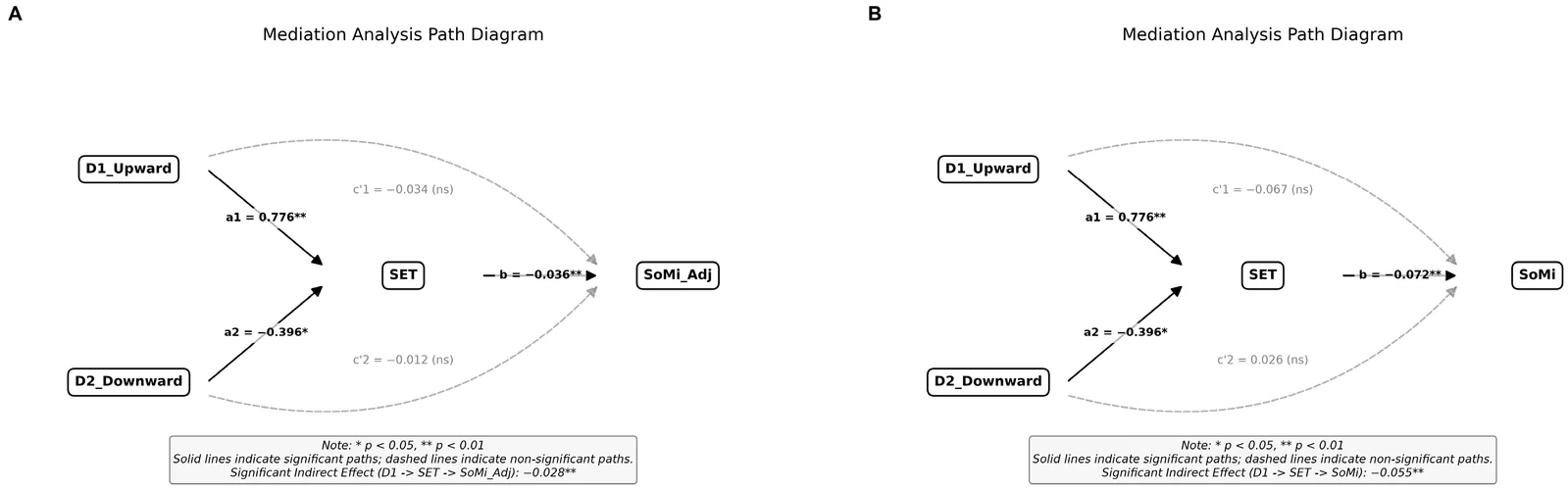
Path diagrams of the mediation analysis depicting the effects of experimental conditions on SoMi_Adj (**A**) and SoMi (**B**) through the mediator SET. Note: The figure illustrates the mediation models with Upward (D1) and Downward (D2) conditions as independent variables (Control group as reference), SET as the mediator, and SoMi_Adj (Panel **A**) and SoMi (Panel **B**) as dependent variables. Values on the paths represent unstandardized regression coefficients. ns, not significant.

Subsequently, we fitted a multicategorical mediation model, with the control group as the reference category, to examine the indirect association between social comparison condition and social mindfulness through self-evaluation threat. With the control group as the reference, upward comparison was associated with higher self-evaluation threat (*B* = 0.776, bootstrap *SE* = 0.235, *p* = 0.001), whereas downward comparison was associated with lower self-evaluation threat (*B* = −0.396, bootstrap *SE* = 0.186, *p* = 0.033; Figure 6A). Higher self-evaluation threat was associated with lower preference-adjusted SoMi after accounting for comparison condition (*B* = −0.036, bootstrap *SE* = 0.008, *p* < 0.001).

Bootstrap analysis confirmed a significant relative indirect effect for the “Upward Comparison → Self-Evaluation Threat → Social Mindfulness” pathway (indirect effect = −0.028, 95% CI [−0.050, −0.010]). After controlling for SET, the direct effect of upward comparison on social mindfulness was not statistically significant (*β* = −0.034, *p* = 0.224). These findings support an indirect association between upward comparison and social mindfulness through self-evaluation threat. Analyses using classical SoMi scores yielded a similar pattern of indirect effects (indirect effect = −0.055, 95% CI [−0.100, −0.020]; see Figure 6B).

#### 3.2.4. Cognitive Mechanism Validation: Evidence from HDDM

**Figure 7:**
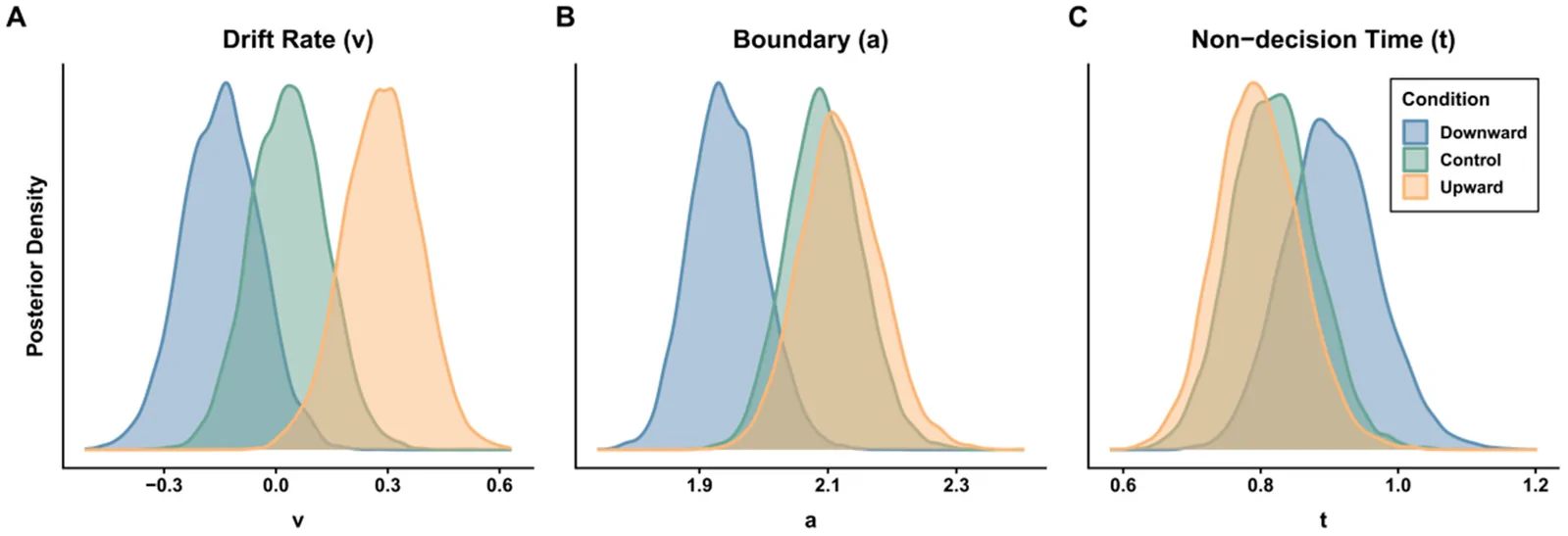
Posterior density distributions of hierarchical drift diffusion model (HDDM) parameters across conditions in Experiment 2. Note: The figure displays the posterior distributions for DDM parameters under Downward (blue), Control (green), and Upward (orange) conditions. (**A**) Drift Rate (*v*), (**B**) Boundary Separation (*a*), (**C**) Non-Decision Time (*t*).

Parameter traces and posterior predictive response-time checks are shown in Supplementary Figures S3 and S4. These checks compare observed and model-predicted response-time distributions overall and by comparison condition. The posterior distributions of drift rate (*v*), boundary separation (*a*), and non-decision time (*t*) across the three comparison conditions in Experiment 2 are presented in Figure 7.

Direct posterior contrasts indicated a higher drift rate under upward comparison than under downward comparison (Δ*v* = 0.430, 95% CrI [0.156, 0.703], P(Δ*v* > 0) = 0.999). The upward–control contrast was less conclusive (Δ*v* = 0.250, 95% CrI [−0.023, 0.518], P(Δ*v* > 0) = 0.964). Boundary separation was also higher under upward than downward comparison on the model’s log scale (Δlog(a) = 0.087, 95% CrI [0.007, 0.168], P(Δlog(a) > 0) = 0.984). All pairwise contrasts for non-decision time had 95% CrIs that included zero. Full pairwise posterior contrasts for Experiment 2 are also reported in Table S1. With the upper response-time cutoff increased to 4.0 s, the upward–downward drift-rate contrast remained positive in Experiment 1 (Δv = 0.428, 95% CrI [0.194, 0.665]) and Experiment 2 (Δv = 0.364, 95% CrI [0.153, 0.579]). Some boundary-separation contrasts were sensitive to the cutoff; the full sensitivity results are reported in Supplementary Material S6.

**Figure 8:**
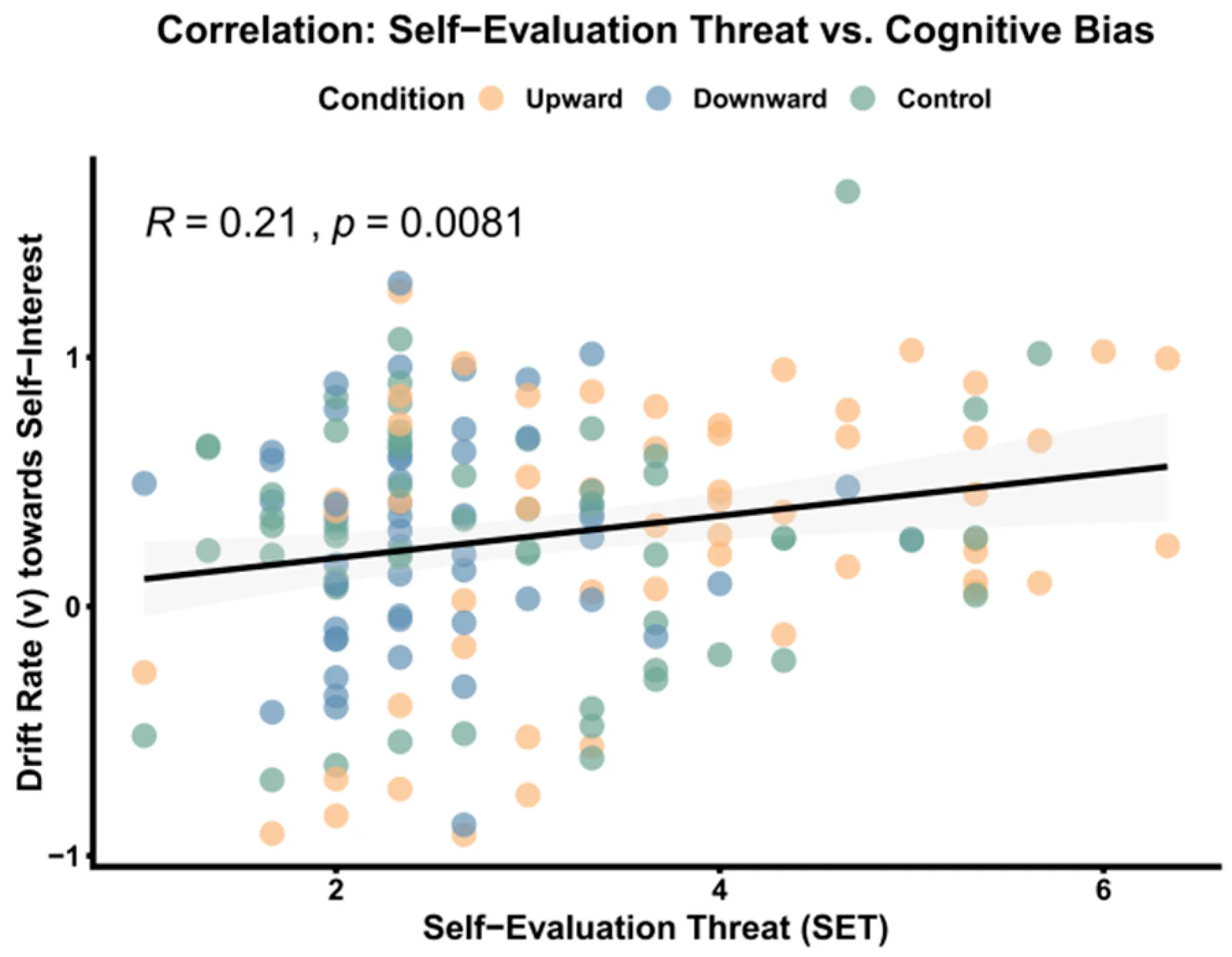
Pearson correlation analysis between Self-Evaluation Threat (SET) and the Drift Rate (*v*) towards self-interest.

**Figure 9:**
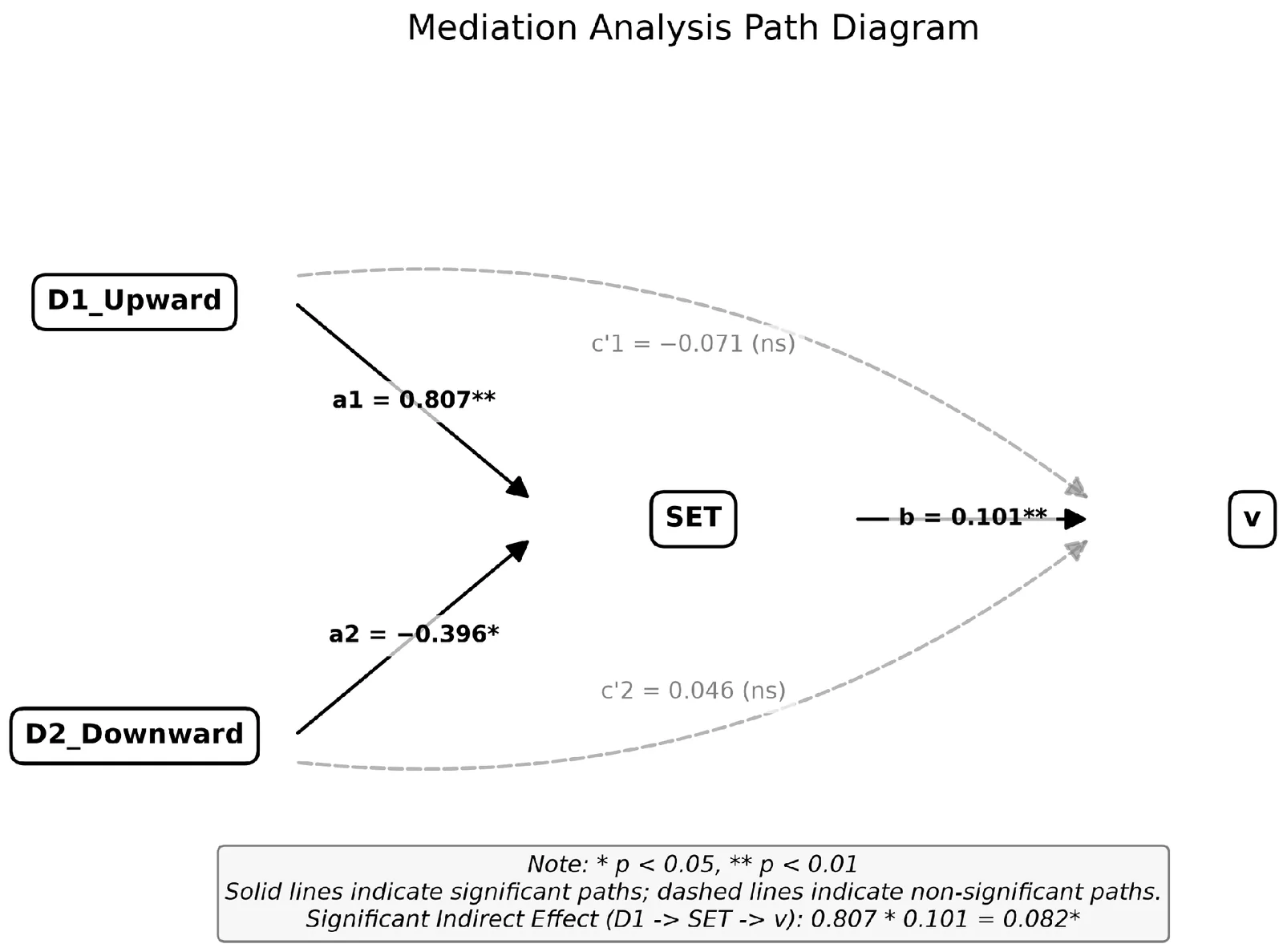
Path diagrams of the mediation analysis depicting the effects of experimental conditions on drift rate parameters (*v*) through the mediator SET. ns, not significant.

The analyses relating self-evaluation threat to individual drift-rate estimates included 163 participants. Finally, we examined the association between self-evaluation threat and the estimated drift rate. Correlation analysis revealed that higher self-evaluation threat was significantly associated with a drift rate more biased toward self-interest (See Figure 8). Structural equation modeling further indicated a significant indirect effect of upward comparison on the estimated drift rate through self-evaluation threat (Indirect Effect = 0.082, 95% CI [0.020, 0.165]; see Figure 9). This finding provides model-based evidence of an association between self-evaluation threat and decision dynamics.

### 3.3. Summary

By combining a virtual comparison paradigm with computational modeling, Experiment 2 replicated the reduction in social mindfulness following upward comparison and examined how self-evaluation threat related to the modeled decision process. The mediation analysis indicated an indirect association between upward comparison and social mindfulness through self-evaluation threat. At the decision-process level, greater threat was associated with a drift rate (*v*) shifted toward selecting the unique item. This pattern is consistent with a change in how evidence favors the two response options. Building on these findings, Experiment 3 examined regulatory focus as a boundary condition, testing whether promotion focus attenuates self-evaluation threat and the behavioral effect of upward comparison.

## 4. Experiment 3

### 4.1. Method

#### 4.1.1. Participants

We recruited 156 undergraduate students majoring in disciplines other than psychology from universities in China. After excluding 12 participants who failed the attention check, the final sample consisted of 144 valid participants (*N_male_* = 55, *N_female_* = 89; *M_age_* = 20.06, *SD* = 1.80). Participants were randomly assigned to a 2 (Social Comparison: Upward vs. Downward) × 2 (Regulatory Focus: Prevention vs. Promotion) between-subjects design. The distribution of valid participants across the four conditions was as follows: 35 in the prevention focus/upward comparison condition, 37 in the prevention focus/downward comparison condition, 36 in the promotion focus/upward comparison condition, and 36 in the promotion focus/downward comparison condition. They provided written informed consent prior to the experiment and received compensation upon completion. A sensitivity analysis conducted during revision evaluated the detectable effect size for the social comparison × regulatory focus interaction. With *N* = 144, four between-subjects conditions, a significance level of 0.05, and a target power of 0.80, the noncentral *F* distribution (numerator *df* = 1; denominator *df* = 140) indicated a minimum detectable effect of Cohen’s f = 0.235, equivalent to partial *η*^2^ = 0.052 under these assumptions. A scenario-based Monte Carlo power analysis for the moderated mediation model was also conducted; its assumptions, procedures, and results are reported in Supplementary Material S8.

#### 4.1.2. Materials

##### Measurement of Trait Social Mindfulness

The protocol was identical to Experiment 1. The Cronbach’s *α* coefficient for the scale was 0.70.

##### Measurement of Trait Regulatory Focus

We assessed dispositional regulatory focus using the Chinese version of the Regulatory Focus Questionnaire (RFQ) revised by Yao et al. (2008). The questionnaire comprises 10 items divided into two subscales: a 4-item prevention focus subscale and a 6-item promotion focus subscale. Items were rated on a 5-point Likert scale ranging from “never” to “always”. In the present study, the Cronbach’s *α* coefficients for the prevention and promotion subscales were 0.73 and 0.64, respectively. The coefficient for the total scale was 0.72, indicating satisfactory reliability.

##### Manipulation and Check of Situational Regulatory Focus

Following established protocols (Friedman & Förster, 2005; Lee et al., 2000), we employed a dual-task paradigm consisting of a self-guidance task and a maze task to prime situational regulatory focus. The effectiveness of the manipulation was verified using a 3-item questionnaire adapted from Pham and Avnet (2004). See Supplementary Material S7 for specific materials. The manipulation check comprised three bipolar items rated on a seven-point scale. The second and third items were reverse-scored, and the three items were averaged so that higher scores indicated a stronger promotion orientation. Internal consistency was Cronbach’s α = 0.69.

##### Social Comparison Manipulation and Check

Identical to Experiment 2.

##### Self-Evaluation Threat Measurement

The same three-item measure and scoring procedure were used immediately after the social comparison manipulation. Internal consistency was Cronbach’s α = 0.78.

##### State Social Mindfulness Measurement

Identical to Experiment 1.

#### 4.1.3. Experimental Procedure

The procedure consisted of five consecutive phases. First, participants provided sociodemographic information and completed the trait social mindfulness scale and trait regulatory focus questionnaire to establish baseline levels. Subsequently, the experiment entered the priming phase for situational regulatory focus. Participants were randomly assigned to either the prevention focus or promotion focus group. Upon completing the self-guidance and maze tasks, they immediately responded to the manipulation check items. The third phase involved the social comparison manipulation. Participants were randomly assigned to either the upward or downward comparison group, where they read the designated materials and completed the virtual character comparison task along with the corresponding manipulation check. Immediately following this, participants were required to complete the self-evaluation threat scale to capture their instantaneous psychological state. Finally, all participants completed the SoMi paradigm task to assess their level of social mindfulness under the different experimental conditions.

Moderated mediation analyses were conducted using PROCESS for SPSS version 3.3 (Model 8), with 5000 bootstrap samples and 95% percentile confidence intervals. Social comparison was coded 1 = upward and 2 = downward comparison; regulatory focus was coded 1 = promotion and 2 = prevention focus. The interaction between social comparison and regulatory focus was included in both the mediator and outcome equations. Self-evaluation threat was the mediator, and preference-adjusted and classical SoMi scores were analyzed separately. No covariates were included.

### 4.2. Results

#### 4.2.1. Extraneous Variables and Manipulation Check

Baseline comparisons revealed no significant differences in trait social mindfulness or trait regulatory focus among the four groups (*p*s > 0.05). Manipulation checks were effective: the upward comparison group perceived their rank as significantly lower than the downward group (*p* < 0.001). For the regulatory-focus manipulation check, participants in the promotion-focus condition (*n* = 72, *M* = 4.81, *SD* = 1.41) scored higher than those in the prevention-focus condition (*n* = 72, *M* = 4.33, *SD* = 1.45), *t*(142) = 2.02, *p* = 0.045, Cohen’s *d* = 0.34. This difference was in the expected direction and provided support for the effectiveness of the regulatory-focus manipulation.

#### 4.2.2. The Moderating Effect of Regulatory Focus on Social Mindfulness

**Figure 10:**
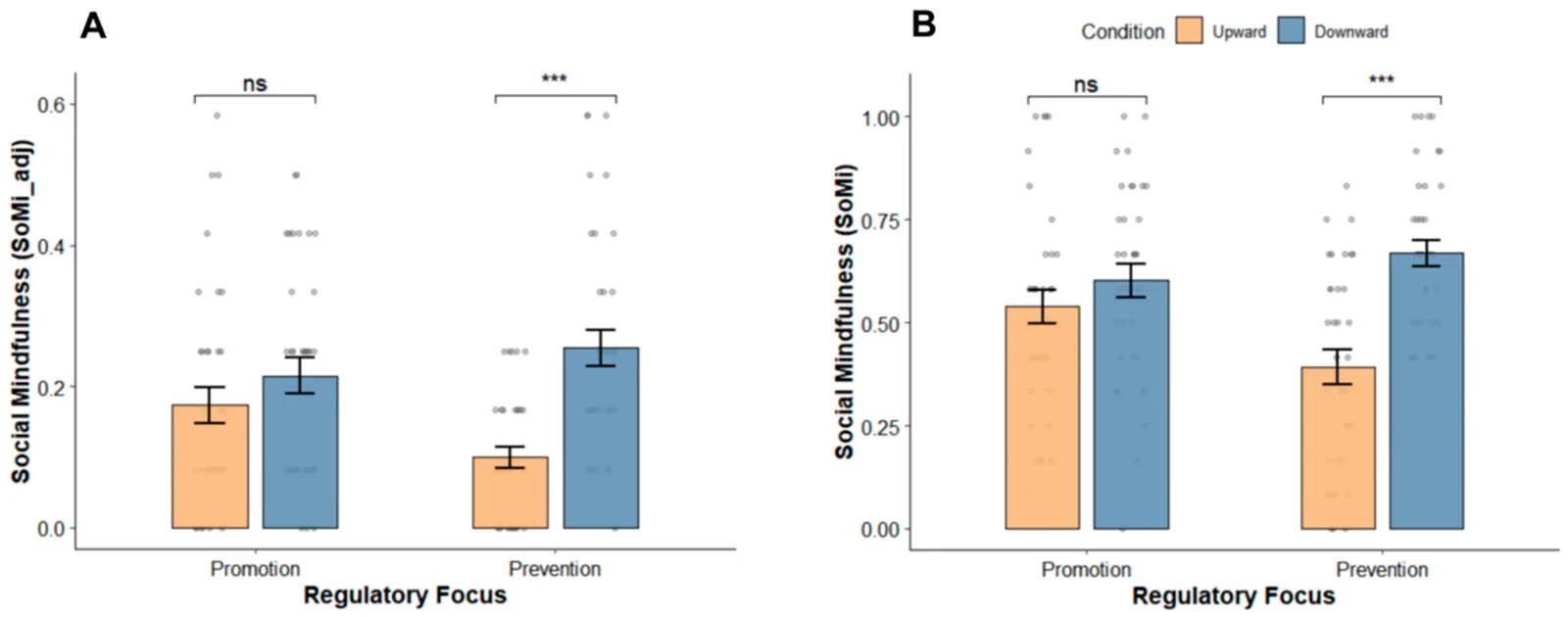
The interactive effects of social comparison and regulatory focus on social mindfulness. (**A**) Adjusted Social Mindfulness scores (SoMi_Adj); (**B**) Standard Social Mindfulness scores (SoMi). Gray dots represent individual participants’ scores; bars represent group means. Significance levels: *** *p* < 0.001; ns indicates non-significance.

A 2 (Social Comparison) × 2 (Regulatory Focus) ANOVA on preference-adjusted SoMi scores yielded (see Figure 10A) a significant interaction effect (*F*(1, 140) = 5.82, *p* = 0.017, *η_p_*^2^ = 0.04). Simple effects analysis revealed that under the prevention focus condition, upward comparison significantly reduced social mindfulness (*M* = 0.10) compared to downward comparison (*M* = 0.25; *F*(1, 140) = 21.80, *p* < 0.001, *η_p_*^2^ = 0.14), consistent with the directional pattern observed in Experiments 1 and 2. However, under the promotion focus condition, the upward–downward difference in social mindfulness was smaller and was not statistically significant (*M*_up_ = 0.17, *M*_down_ = 0.22; *p* = 0.210). Analyses using classical SoMi scores showed a consistent pattern (see Figure 10B and Supplementary Material S2 for detailed statistics of classical SoMi).

#### 4.2.3. Psychological Mechanism: Moderated Mediation

**Figure 11:**
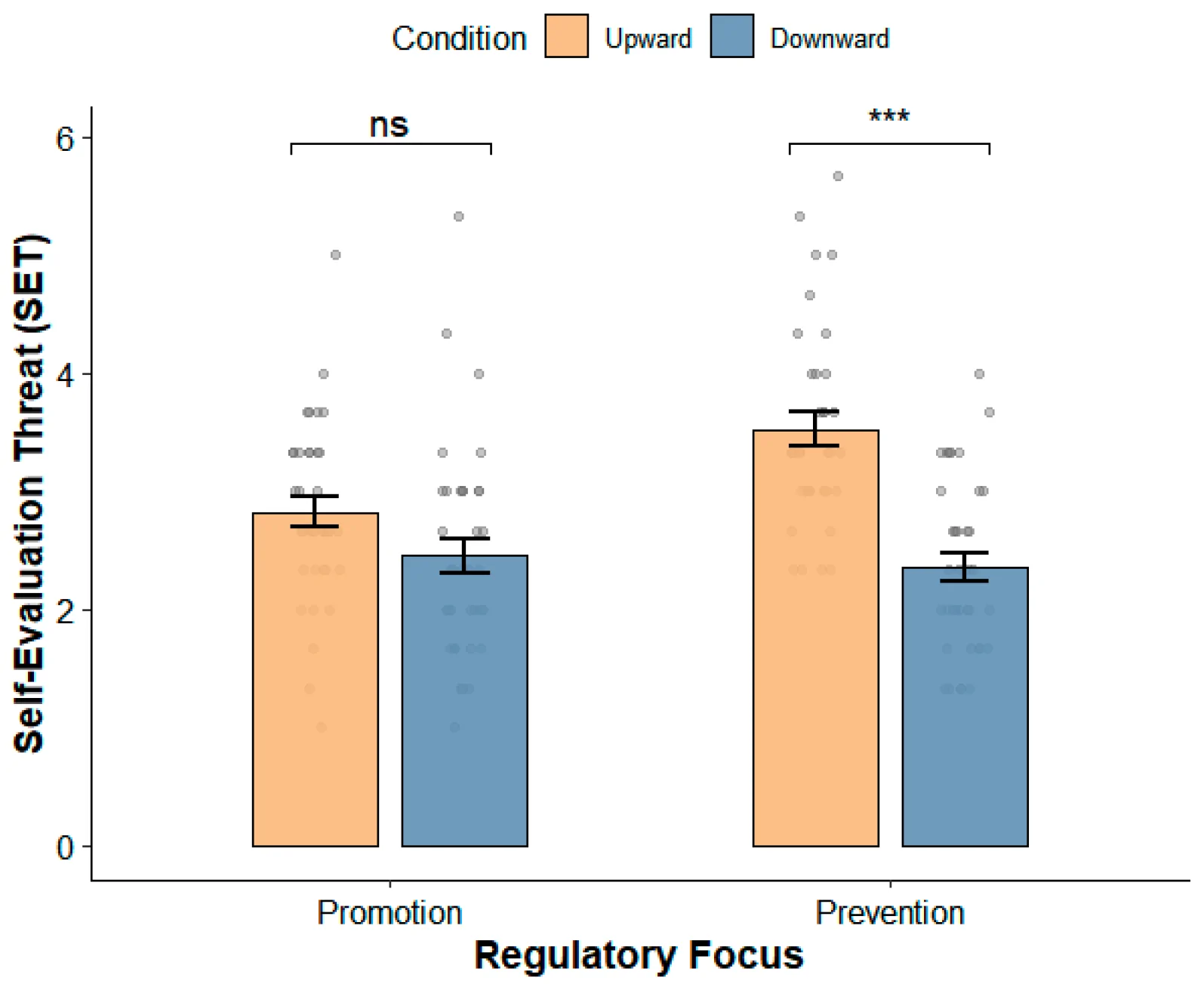
The moderating effect of regulatory focus on the relationship between social comparison and self-evaluation threat. Significance levels: *** *p* < 0.001; ns indicates non-significance.

To elucidate the mechanism underlying this buffering effect, we examined the response of SET. The ANOVA revealed a significant interaction between social comparison and regulatory focus on SET (*F*(1, 140) = 8.60, *p* = 0.004, *η_p_*^2^ = 0.06). Specifically, the upward–downward difference in self-evaluation threat was larger under prevention focus (*p* < 0.001, *η_p_*^2^ = 0.21), whereas the upward–downward difference in self-evaluation threat was smaller under promotion focus and did not reach statistical significance (*p* = 0.057, *η_p_*^2^ = 0.03) (see Figure 11).

**Figure 12:**
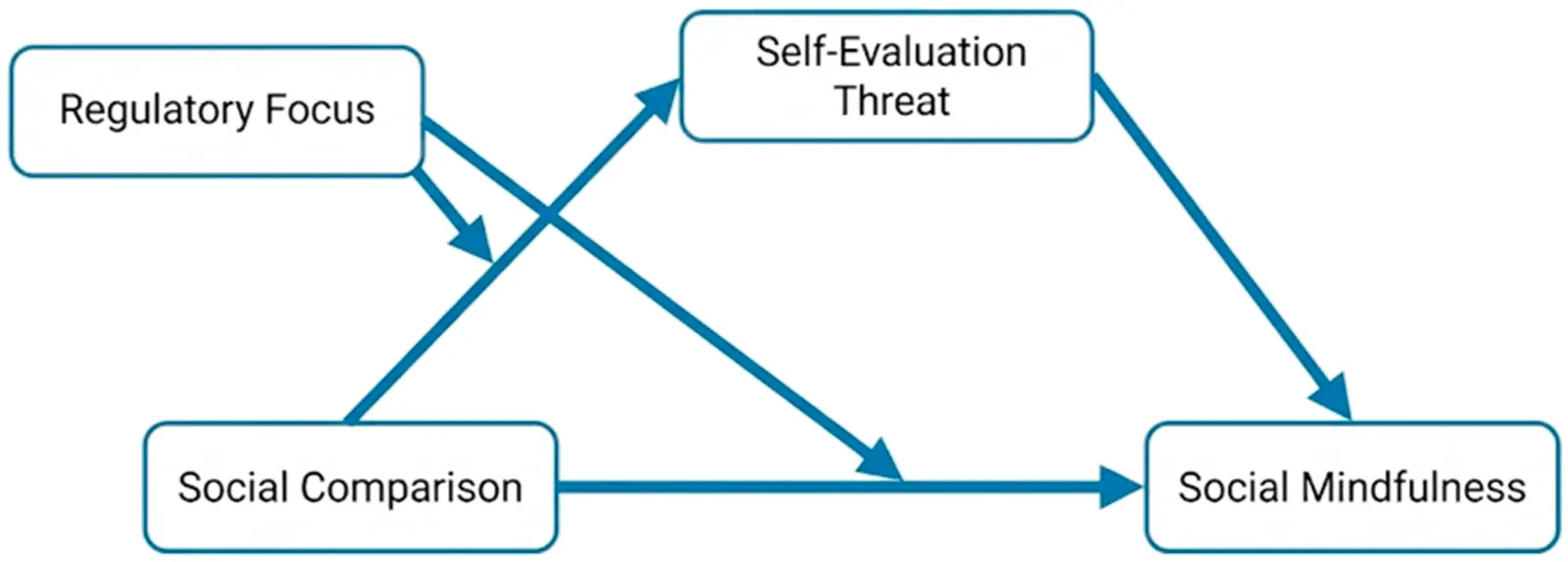
Schematic diagram of the moderated mediation model. Arrows indicate the direction of the paths specified in the model.

The moderated mediation analysis indicated that the indirect association between social comparison and preference-adjusted social mindfulness through self-evaluation threat differed across regulatory-focus conditions (index of moderated mediation = 0.0220, 95% CI [0.0004, 0.0483]; see Figure 12). The conditional indirect effect was 0.0322 under prevention focus (95% CI [0.0009, 0.0704]) and 0.0102 under promotion focus (95% CI [−0.0009, 0.0348]). These findings support a smaller indirect effect under promotion focus, rather than the elimination of the pathway. Analyses using the classical SoMi score yielded a consistent pattern.

### 4.3. Summary

Experiment 3 identified regulatory focus as a boundary condition for the effect of social comparison on social mindfulness. Under prevention focus, upward comparison was associated with greater self-evaluation threat and lower social mindfulness than downward comparison. Under promotion focus, these differences were smaller, and the difference in social mindfulness was not statistically significant. The moderated mediation analysis further indicated that the indirect association through self-evaluation threat varied by regulatory focus. These findings suggest that promotion focus may attenuate the behavioural consequences of upward comparison. A change in how participants appraised the superior target is one possible explanation.

## 5. General Discussion

Across three experiments integrating behavioral paradigms, psychometrics, and the HDDM, this study systematically investigated how academic social comparison affects social mindfulness and examined the roles of self-evaluation threat and regulatory focus. Classical SoMi scores showed an asymmetric pattern: upward comparison was associated with lower scores than downward comparison or the control condition, whereas downward comparison did not increase scores relative to control. Preference-adjusted scores followed the same direction, although the post hoc pairwise differences were not statistically significant after Holm correction. HDDM analyses in Experiments 1 and 2 further identified a drift-rate shift toward the unique-item response under upward comparison, providing a model-based characterization of the observed choice differences.

Specifically, Experiment 2 showed that upward comparison increased self-evaluation threat, and greater threat was associated with both lower social mindfulness and a drift rate more strongly oriented toward the unique-item choice. This pattern is consistent with the possibility that threat changes how information about the available options influences the decision. Experiment 3 further showed that promotion focus attenuated the upward–downward differences in threat and social mindfulness. Together, the behavioural and modelling findings provide a basis for considering the motivational and cognitive accounts discussed below.

### 5.1. Upward Social Comparison, Self-Evaluation Threat, and Social Mindfulness

Our study found an asymmetric pattern in the classical SoMi scores. In both experiments, these scores were lower following upward comparison than following downward or no comparison. Preference-adjusted scores showed the same directional pattern, although their post hoc pairwise differences were not statistically significant after Holm correction. This finding aligns with the results of Schlosser and Levy (2016) and Gong and Sanfey (2017), indicating that social comparison placing an individual in a disadvantaged position suppresses prosocial behavior toward strangers.

We can trace the deep-seated drivers of this behavior to the Self-Evaluation Maintenance (SEM) model (Tesser, 1988). SEM theory posits that individuals possess an intrinsic need to maintain a positive self-evaluation. When individuals engage in upward comparison in a self-relevant domain, such as academic performance, the perceived disparity directly strikes at their sense of self-worth. Consistent with this account, upward comparison increased self-evaluation threat in Experiment 2, and the mediation analysis indicated a significant indirect association between comparison condition and social mindfulness through threat. Research on malicious envy suggests that unfavorable upward comparisons can, under some conditions, elicit hostile motivations toward advantaged others (Crusius & Lange, 2021). Related work also links upward comparison to reduced perceived control and compensatory choices in another behavioral domain (Che et al., 2025). These findings offer possible explanations for a more self-focused response to comparison, but the present study did not measure envy or compensatory motivation. In the SoMi task, the person whose options were restricted was also not the superior comparison target.

Furthermore, from an evolutionary psychology perspective, upward comparison may heighten awareness of one’s relative competitive standing (Garcia et al., 2013; Swencionis & Fiske, 2020). Research on social hierarchy distinguishes dominance and prestige as different strategies related to gaining and maintaining social rank (McClanahan et al., 2022). This literature raises the possibility that concerns about relative standing shaped participants’ subsequent choices.

Notably, downward comparison did not significantly enhance social mindfulness relative to the control condition. This finding suggests that the behavioural consequences of upward and downward comparison may be asymmetric: a more favourable relative position does not necessarily translate into greater consideration of another person’s choice options. The pattern is consistent with self-evaluation threat contributing to the reduction associated with upward comparison, without establishing it as the sole process involved (Van Doesum et al., 2025).

### 5.2. Psychological Threat and Cognitive Decision Processes

The most significant theoretical innovation of this study lies in the integration of the HDDM, which provides a model-based account of the decision processes associated with social comparison. The modeling results from Experiments 1 and 2 consistently showed a drift-rate (v) shift toward selecting the unique item under upward comparison. The pattern for boundary separation (a) differed across experiments. In Experiment 1, boundary separation was lower under downward comparison than in the control condition, whereas the upward–downward and upward–control contrasts had 95% credible intervals that included zero. In Experiment 2, boundary separation was higher under upward than downward comparison, while the remaining contrasts had intervals that included zero. For non-decision time (t), all pairwise contrasts had 95% credible intervals that included zero in both experiments.

The drift rate reflects the direction and average rate of evidence accumulation within the fitted model (Wiecki et al., 2013). In the upward comparison group, the estimated drift rate shifted toward selecting the unique item, which left fewer options for the subsequent chooser. Experiment 2 further showed that greater self-evaluation threat was associated with a drift rate more strongly oriented toward the unique-item choice. This pattern is consistent with threat altering how information relevant to the two options influences the decision. Threat-related cues have also been found to affect evidence accumulation in perceptual tasks (Ozturk et al., 2024).

One possible explanation can be drawn from the Strength Model of Self-Control (Baumeister et al., 1998). Responding to self-evaluation threat may draw on resources that could otherwise be directed toward considering another person’s options. Social mindfulness involves recognizing how one’s choice affects what remains available to the subsequent chooser (Van Doesum et al., 2013). From this perspective, heightened concern with one’s own standing may reduce the weight given to that consequence when making a choice.

A related explanation draws on the focusing effect described by Muller and Butera (2007): self-evaluation threat can narrow attention toward information relevant to one’s own evaluation. In the SoMi task, preserving the partner’s choice requires noticing that selecting the unique item would leave the partner with only one type of object, and taking that consequence into account when choosing (Van Doesum et al., 2013). Following upward comparison, a stronger focus on one’s own standing may make this consequence less salient, increasing the relative influence of the unique-item option during evidence accumulation. This account is consistent with the drift-rate shift observed in our study. The same shift could also occur if participants noticed the partner’s options but placed less value on preserving them.

### 5.3. The Moderating Role of Promotion Focus

Given the association between self-evaluation threat and social mindfulness in Experiment 2, Experiment 3 examined whether regulatory focus moderated the effect of upward comparison. The results of Experiment 3 indicate that regulatory focus acts as a critical boundary condition for the social comparison effect. Under the prevention focus condition, upward comparison was associated with greater self-evaluation threat and lower social mindfulness than downward comparison. However, under the promotion focus condition, the upward–downward differences were smaller, and the difference in social mindfulness was not statistically significant. This finding extends the application of Regulatory Focus Theory to the domain of prosociality and supports the theoretical expectations regarding the relationship between motivation and social comparison proposed by Higgins (1997) and Fayant et al. (2011).

Specifically, distinct regulatory foci trigger fundamentally different modes of cognitive evaluation and processing. Prevention focus centers on safety and duty. This avoidance motivation renders individuals hypersensitive to negative information (Hamamura et al., 2009). A superior comparison target may therefore be more likely to be construed as a potential threat or a sign of falling short under prevention focus. Such an appraisal could intensify self-evaluation threat and contribute to the larger behavioural difference observed in this condition (Sherman & Cohen, 2006).

In contrast, promotion focus centers on growth and aspirations (Higgins, 1997). This orientation may encourage individuals to consider what they could learn from a superior comparison target, rather than viewing the performance gap solely as evidence of their own inadequacy. Previous research suggests that promotion-focused individuals are particularly responsive to positive role models (Lockwood et al., 2002), and that upward comparison can support engagement in some learning contexts (Neugebauer et al., 2016). Research on social comparison also identifies assimilation as a possible response to a superior target, particularly in approach-oriented contexts (Fayant et al., 2011; Suls & Wheeler, 2000). Applied to the present study, such an interpretation could make the superior student appear more attainable and help explain the smaller upward–downward difference in self-evaluation threat under promotion focus.

These differences in motivational orientation may carry over to the subsequent choice task. When upward comparison is experienced as less threatening, concerns about one’s own standing may become less prominent, leaving greater scope to consider the options available to the next chooser. This account is consistent with the smaller upward–downward differences in self-evaluation threat and social mindfulness under promotion focus. The moderated mediation result further indicates that the indirect association through self-evaluation threat varied by regulatory focus. Together, these findings suggest that promotion focus may attenuate the interpersonal consequences of upward comparison.

### 5.4. Limitations and Future Directions

This study is subject to several limitations. First, all participants were recruited from universities in China, and the social comparisons focused on academic performance. Future research should examine whether the findings extend to other comparison domains, such as workplace achievement or financial standing. Cultural context may also shape how people interpret academic comparisons and how much importance they attach to preserving another person’s choice options. Previous research has reported cultural variation in social-comparison seeking (White & Lehman, 2005) and cross-national variation in social mindfulness choices (Van Doesum et al., 2021). The present study cannot determine whether its findings would differ across cultures. Future studies could use comparable comparison manipulations and SoMi tasks with student samples from China and selected Western countries. If the Chinese-validated SMSRS is used for such comparisons, its measurement equivalence across language groups should first be examined. Second, the HDDM findings provide model-based inferences about decision processes rather than direct measurements of attention or neural processing. A drift-rate difference does not distinguish reduced cognitive capacity to process another person’s options from differences in attentional weighting, preference, or motivational valuation. Future studies could combine the SoMi task with eye tracking, direct valuation measures, and electroencephalography (EEG) or functional magnetic resonance imaging (fMRI) to distinguish these accounts and examine their relationship with the estimated decision parameters.

Third, the reverse-scored self-evaluation composite provided an indirect indicator of self-evaluation threat, but its specificity relative to state self-esteem and perceived competence was not established. Future studies should include separate measures of these constructs and direct assessments of perceived threat to examine their convergent and discriminant validity. Some measures also showed modest internal consistency, including the trait promotion-focus subscale (Cronbach’s α = 0.64) and the situational regulatory-focus manipulation check (α = 0.69). Measurement error may have reduced the precision of the baseline comparisons and the assessment of manipulation effectiveness. Future studies should use measures with stronger reliability to assess these constructs. Finally, this study measured social mindfulness within online virtual interactions. Future research should attempt to validate these conclusions in offline, face-to-face interaction contexts which possess higher ecological validity.

## 6. Conclusions

In Experiments 1 and 2, classical SoMi scores were lower under upward academic comparison than under downward comparison or the no-comparison control condition. Preference-adjusted scores showed the same directional pattern, but the post hoc pairwise differences were not statistically significant after Holm correction. Experiment 2 showed that upward comparison increased self-evaluation threat and that greater threat was associated with lower social mindfulness, with a significant indirect effect in the mediation analysis. These results place self-evaluation threat among the processes relevant to the behavioural effect of upward comparison.

The HDDM analyses in Experiments 1 and 2 further showed a drift-rate shift toward selecting the unique item under upward comparison. This finding adds a model-based account of decision dynamics to the behavioural results. In Experiment 2, boundary separation also differed between the upward and downward comparison conditions. The drift-rate pattern is compatible with the attentional, valuation-based, preference-related, and motivational interpretations discussed above.

Experiment 3 showed that promotion focus attenuated the upward–downward differences in self-evaluation threat and social mindfulness. This result identifies regulatory focus as a boundary condition and suggests that how an upward comparison is approached may matter for subsequent interpersonal choices. Interpreting a superior target as an attainable role model is one possible account of this pattern. Further research could test that appraisal directly and examine whether regulatory focus also changes HDDM parameters. Together, the findings connect social comparison, self-evaluation threat, observed choice, and modelled decision dynamics, with potential relevance to competitive educational and organizational settings.

## Data Availability

The de-identified data supporting the findings of this study are available from the corresponding author upon reasonable request. The data are not publicly available due to privacy and ethical restrictions.

## Supplementary Materials

The following supporting information can be downloaded at: https://www.mdpi.com/article/10.3390/bs16101856/s1, Supplementary Materials S1–S9 (Donnelly et al., 2023), including Figures S1–S4 and Tables S1–S4. Figure S1: MCMC trace plots for HDDM parameters in Experiment 1; Figure S2: posterior predictive response-time checks for the HDDM in Experiment 1; Figure S3: MCMC trace plots for HDDM parameters in Experiment 2; Figure S4: Posterior predictive response-time checks for the HDDM in Experiment 2; Table S1: pairwise posterior contrasts of HDDM parameters in Experiments 1 and 2; Table S2: reference regression coefficients used to calibrate the Experiment 3 simulation scenarios; Table S3: estimated power for the index of moderated mediation at N = 144; and Table S4: quasi-binomial robustness tests for preference-adjusted SoMi.
