# Supplementary material for: Academic Social Comparison and Social Mindfulness: The Roles of Self-Evaluation Threat and Regulatory Focus
Source: Behav Sci (Basel). 2026 Oct 9;16(10):1856. doi: 10.3390/bs16101856 (PMC13701830; doi:10.3390/bs16101856)
Supplement: Supplementary file 1 [file behavsci-16-01856-s001.zip › behavsci-4569436-supplementary.pdf]

Supplementary Materials S1

Preliminary Analyses and Manipulation Checks in Experiment 1

To rule out the influence of confounding variables, we first examined the objective academic ranking, the subjective importance of grades, and trait social mindfulness of the participants. Independent samples t-tests revealed no significant difference in objective academic ranking between the upward and downward comparison groups ($t(110) = -0.47$, $p = 0.640$). Similarly, no significant difference was found regarding the importance of grades ($t(110) = -0.64$, $p = 0.524$). A one-way analysis of variance (ANOVA) indicated no significant differences in trait social mindfulness among the three groups ($F(2, 164) = 0.49$, $p = 0.611$). This suggests that the groups were homogeneous at baseline.

As the no-comparison control group did not involve comparative information, they were exempt from the manipulation check items. An independent samples t-test showed that participants in the downward comparison group perceived their academic performance as significantly better than their comparison target ($M_{down} = 4.59$, $SD_{down} = 0.89$) compared to the upward comparison group ($M_{up} = 1.54$, $SD_{up} = 1.04$; $t(110) = -16.66$, $p < 0.001$). This confirms the effectiveness of the social comparison manipulation.

Supplementary Materials S2

1. Robustness Check: The Effect of Social Comparison on Classical Social Mindfulness Scores (Experiment 1)

We conducted a one-way ANOVA with social comparison condition as the independent variable and classical SoMi score as the dependent variable. The analysis showed a significant condition effect (M_{up} = .34, SD_{up} = .28; M_{down} = .52, SD_{down} = .25; M_{control} = .52, SD_{control} = .28), $F(2, 164) = 8.88$, $p < .001$, $\eta p^2 = .10$ (see Figure 2A). Post hoc comparisons with Holm correction showed lower scores in the upward group than in the downward group ($t(110) = -3.74$, adjusted $p < .001$, Cohen's $d = .71$) and the control group ($t(109) = -3.51$, adjusted $p = .0013$, Cohen's $d = .67$). The downward and control groups did not differ significantly ($t(109) = 0.02$, adjusted $p = .983$).

2. Robustness Check: The Effect of Social Comparison on Classical Social Mindfulness Scores (Experiment 2)

We repeated the one-way ANOVA using classical SoMi score as the dependent variable. The overall condition effect was significant, $F(2, 161) = 8.10$, $p < .001$, $\eta p^2 = .091$ (see Figure 4A). Post hoc comparisons with Holm correction showed lower scores in the upward group than in the downward group ($t(107) = -4.01$, adjusted $p < .001$, Cohen's $d = .77$) and the control group ($t(108) = -2.48$, adjusted $p = .030$, Cohen's $d = .47$). The downward–control difference was not statistically significant ($t(107) = 1.33$, adjusted $p = .186$).

3. Robustness Check: The Moderating Effect of Regulatory Focus on Social Mindfulness (Experiment 3)

We repeated the analysis using the classical SoMi score as the dependent variable. The pattern of results was highly consistent with the primary findings. The interaction effect remained significant ($F(1, 140) = 7.66$, $p = 0.006$, $\eta_p^2 = 0.05$). Simple-effects analysis showed lower social mindfulness following upward than downward comparison under prevention focus ($p < .001$). Under promotion focus, the upward–downward difference was smaller and did not reach statistical significance ($p = .254$). This pattern was consistent with the primary analysis based on preference-adjusted SoMi scores.

Supplementary Materials S3

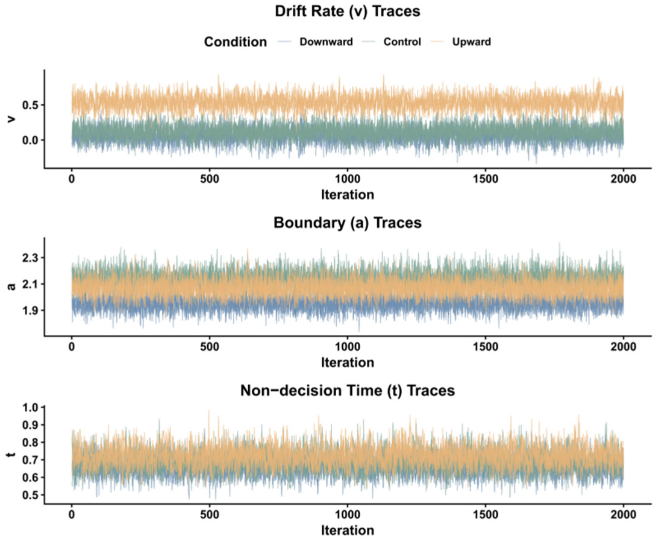

Figure S1. Trace plots of MCMC sampling chains for model parameter estimation in Experiment 1. Note: The trace plots show the sampling history over 2000 iterations for Drift Rate (*v*), Boundary (*a*), and Non-decision Time (*t*), confirming model convergence.

Supplementary Materials S4

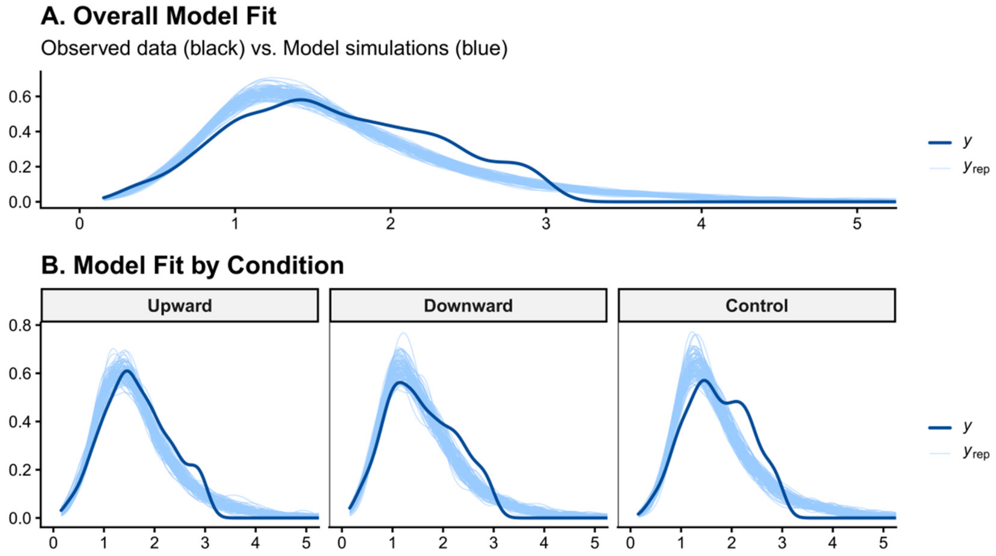

Figure S2. Posterior predictive checks (PPC) for the HDDM in Experiment 1. Note:Comparison between observed data (black lines) and model-predicted data (colored lines) across conditions.

Supplementary Materials S5

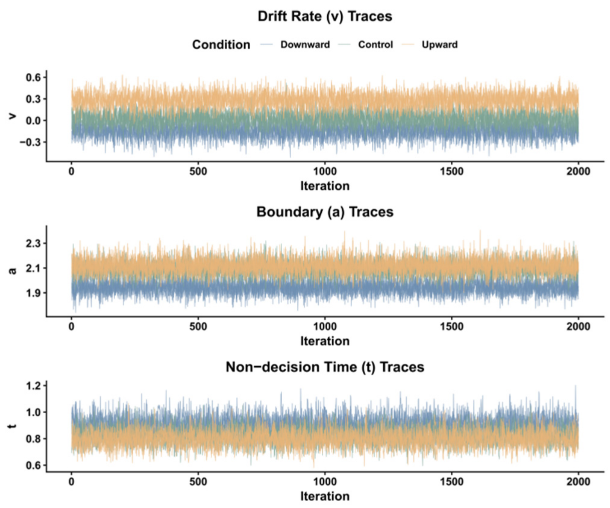

Figure S3. Trace plots of MCMC sampling chains for model parameter estimation in Experiment 2.

Supplementary Materials S6

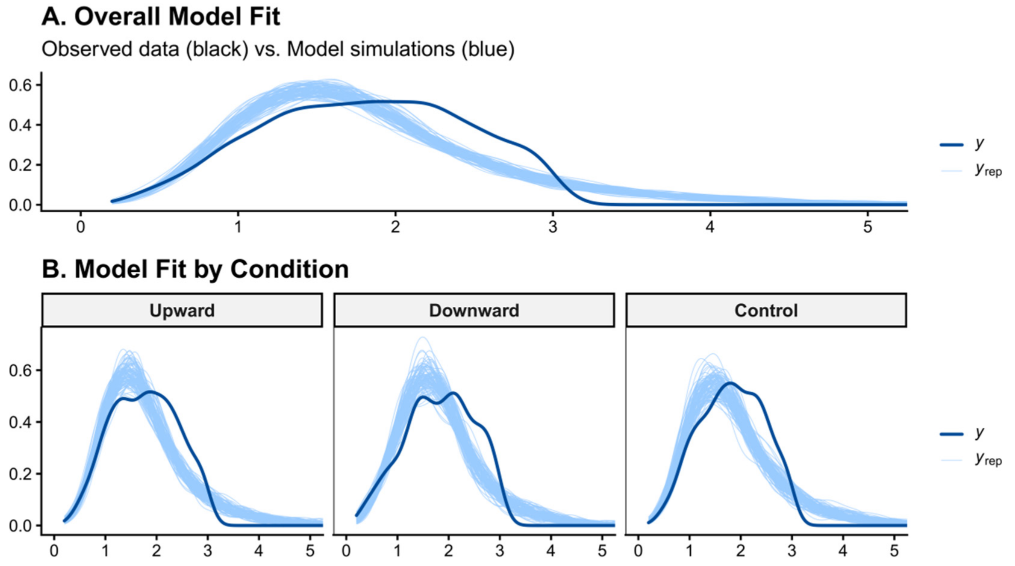

Figure S4. Posterior predictive checks (PPC) for the HDDM in Experiment 2.

HDDM model specification and trial exclusions

In both experiments, the 12 odd-numbered SoMi trials were the experimental trials entered into the decision model; control trials were not included. Responses were coded 1 for the unique item and 0 for a non-unique item. Experimental-trial response times were converted from milliseconds to seconds. Responses at or below 0.3 seconds and at or above 3.0 seconds were excluded. In Experiment 1, 43 trials fell at or below the lower threshold and 475 at or above the upper threshold, leaving 1,486 of 2,004 experimental trials from 167 participants. In Experiment 2,

the corresponding counts were 16 and 629, leaving 1,323 of 1,968 experimental trials contributed by 163 of the 164 participants. The participant with no eligible trials was in the upward-comparison group; this participant remained in the behavioral and self-evaluation-threat analyses. No experimental trial had a missing recorded response or response time in the supplied modelling data.

The models were fitted with the Wiener family in brms 2.23.0 using CmdStan 2.37.0. Condition effects were specified for drift rate (v), boundary separation (a), and non-decision time (t), with upward comparison as the reference group. The starting-point parameter (z) did not vary by condition; participant-level intercepts were included for all four parameters. Boundary separation and non-decision time used log links, and starting-point bias used a logit link. A normal(0, 2) prior was specified for the population-level coefficients, normal(1, 0.5) for the boundary-separation intercept, and normal(−2, 0.5) for the non-decision-time intercept. The remaining priors were Student-t(3, 1.6, 2.5) for the drift-rate intercept in Experiment 1 and Student-t(3, 1.8, 2.5) in Experiment 2, logistic(0, 1) for the starting-point-bias intercept, half-Student-t(3, 0, 2.5) for participant-level standard deviations, and LKJ(1) for correlations among participant-level effects. Four chains of 4,000 iterations were run, with 2,000 warm-up iterations per chain. The adapt_delta settings were 0.99 in Experiment 1 and 0.999 in Experiment 2; maximum tree depth was 15 in both.

Convergence was assessed using chain trace plots and $\hat{R}$ values, with $\hat{R} < 1.01$ used as the criterion for the reported population-level parameters. These parameters met the criterion in both experiments. The trace and posterior predictive figures assess chain behaviour and response-time fit, respectively. Because the posterior predictive figures show overall and condition-level response-time densities, they cannot establish fit to observed choice proportions or to the response-time distribution for each response category. The saved 3-second model draws contained two divergent transitions in Experiment 1 and four in Experiment 2; one iteration reached the maximum tree depth in Experiment 2.

Table S1. Pairwise posterior contrasts of HDDM parameters in Experiments 1 and 2.

Experiment	Parameter	Contrast	Posterior mean difference	95% CrI	P(Δ > 0)
1	Drift rate (v)	Upward − Downward	0.470	[0.193, 0.751]	.999
1	Drift rate (v)	Upward − Control	0.410	[0.132, 0.695]	.997
1	Drift rate (v)	Downward − Control	−0.061	[−0.350, 0.223]	.338
1	Boundary separation, log(a)	Upward − Downward	0.059	[−0.023, 0.145]	.919
1	Boundary separation, log(a)	Upward − Control	−0.033	[−0.118, 0.054]	.215
1	Boundary separation, log(a)	Downward − Control	−0.093	[−0.174, −0.011]	.014

1	Non-decision time, log(t)	Upward − Downward	0.089	[−0.138, 0.309]	.790
1	Non-decision time, log(t)	Upward − Control	0.045	[−0.187, 0.277]	.648
1	Non-decision time, log(t)	Downward − Control	−0.044	[−0.273, 0.184]	.356
2	Drift rate (v)	Upward − Downward	0.430	[0.156, 0.703]	.999
2	Drift rate (v)	Upward − Control	0.250	[−0.023, 0.518]	.964
2	Drift rate (v)	Downward − Control	−0.180	[−0.448, 0.083]	.091
2	Boundary separation, log(a)	Upward − Downward	0.087	[0.007, 0.168]	.984
2	Boundary separation, log(a)	Upward − Control	0.012	[−0.064, 0.090]	.619
2	Boundary separation, log(a)	Downward − Control	−0.075	[−0.150, 0.003]	.031
2	Non-decision time, log(t)	Upward − Downward	−0.125	[−0.330, 0.083]	.113
2	Non-decision time, log(t)	Upward − Control	−0.027	[−0.232, 0.177]	.394
2	Non-decision time, log(t)	Downward − Control	0.098	[−0.108, 0.308]	.826

Note. Contrasts were calculated draw by draw from 8,000 retained posterior samples per model. Each difference (Δ) represents the first condition minus the second condition. CrI = equal-tailed credible interval, defined by the 2.5th and 97.5th percentiles of the posterior contrast distribution. Boundary-separation and non-decision-time contrasts were calculated on the model's log scale. P(Δ > 0) denotes the proportion of posterior draws in which the contrast was positive; it is a posterior directional probability, not a frequentist p-value. Starting-point bias was not allowed to vary by condition and therefore was not included in these contrasts.

Sensitivity to the upper response-time cutoff. We refitted the original models using an upper cutoff of 4.0 seconds; all other trial-coding rules, model specifications, and priors were retained. The analysis included 1,697 trials from 167 participants in Experiment 1 and 1,604 trials from 164 participants in Experiment 2. The upward–downward drift-rate contrast remained positive in Experiment 1 (Δv = .428, 95% CrI [.194, .665]) and Experiment 2 (Δv = .364, 95% CrI [.153, .579]). The upward–control contrast was also positive in the 4-second models (Experiment 1: Δv = .387, 95% CrI [.135, .634]; Experiment 2: Δv = .272, 95% CrI [.061, .477]). The corresponding 3-second interval in Experiment 2 included zero (Table S1), so the strength of evidence for this particular contrast differed across cutoffs. Some boundary-separation contrasts were also cutoff-sensitive: the 3-second credible intervals for downward–control in Experiment 1

and upward–downward in Experiment 2 excluded zero, whereas their 4-second intervals included zero. All non-decision-time contrast intervals included zero under both cutoffs. For the population-level condition coefficients in the 4-second models, all $\hat{R}$ values were below 1.01. There were two divergent transitions in Experiment 1 and none in Experiment 2, with no maximum-tree-depth hits.

Supplementary Material S7

1.Priming Materials for Prevention Focus

(1) In our daily lives, everyone possesses certain duties, responsibilities, and obligations (i.e., things we feel we must do, such as finding a job after graduation or caring for parents). Please recall and list 2 duties or obligations you currently have, and 2 you had during your childhood (please ensure 2 items are listed for each category) in the spaces below.

Past: ① ____________________ ② ____________________

Present: ① ____________________ ② ____________________

(2) Next, please complete a maze task. Scenario: A hungry owl is hovering above the maze, preparing to swoop down and eat the mouse located in the center. If the mouse does not escape immediately, it will be killed by the owl's attack. Task: Please draw a path to help the mouse escape the maze and avoid the owl.

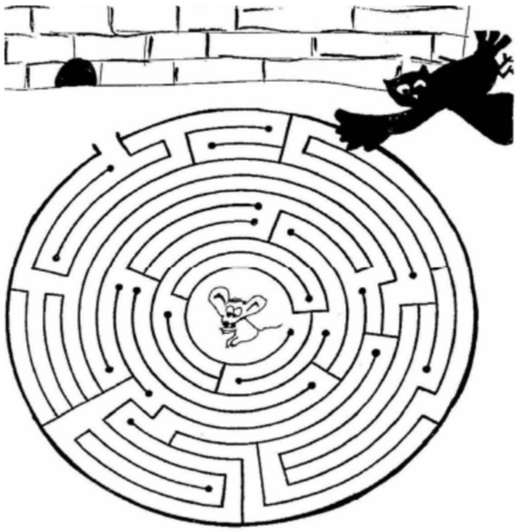

2.Priming Materials for Promotion Focus

(1) In our daily lives, everyone possesses certain hopes, dreams, and aspirations (i.e., things we want to pursue or the person we want to become). Please recall and list 2 hopes or aspirations you currently have, and 2 you had during your childhood (please ensure 2 items are listed for each category) in the spaces below.

Past: ① ____________________ ② ____________________

Present: ① ____________________ ② ____________________

(2) Next, please complete a maze task. Scenario: A piece of delicious cheese is placed at the exit of the maze, and a hungry mouse is located in the center. If the mouse eats the cheese, it will feel very happy. Task: Please draw a path to help the mouse navigate out of the maze to eat the cheese.

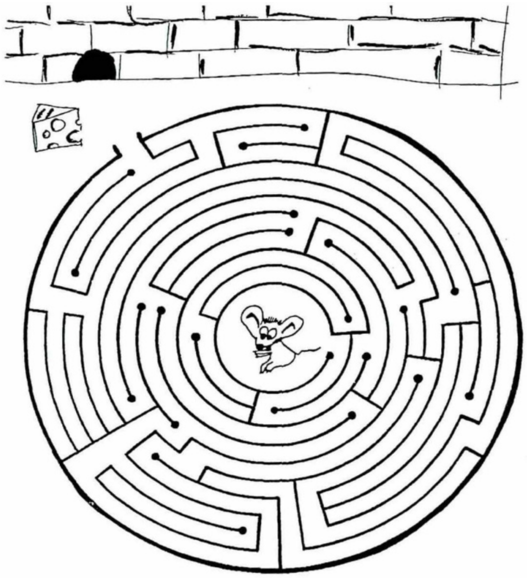

3.Manipulation Check for Situational Regulatory Focus

Please complete the following 3 items. Each item consists of two opposing statements. "1" indicates strong agreement with the statement on the left, and "7" indicates strong agreement with the statement on the right. Other numbers represent varying degrees of agreement. Please circle the number that best corresponds to your current feelings.

(1) I would prefer to do what is generally considered correct. 1 2 3 4 5 6 7 I would prefer to do what I want to do.

(2) I would prefer to travel the world. 1 2 3 4 5 6 7 I would prefer to pay off my debts/loans.

(3) I would prefer to go anywhere I want to go. 1 2 3 4 5 6 7 I would prefer to fulfill any commitment I have made.

Supplementary Material S8: Sensitivity and simulation-based power analyses for Experiment 3

Interaction sensitivity

We conducted a sensitivity analysis for the social comparison × regulatory focus interaction in the 2 × 2 between-subjects design. The calculation used the noncentral F distribution with numerator $df = 1$, denominator $df = 140$, and noncentrality parameter $\lambda = Nf^2$. With $N = 144$, $\alpha = .05$, and target power = .80, the minimum detectable effect was Cohen's $f = .235$ (partial $\eta^2 = .052$). For $f = .10$, .15, .20, .25, and .30, power was .222, .432, .664, .846, and .947, respectively.

Moderated mediation model and simulation assumptions

We conducted a scenario-based Monte Carlo analysis for the model structure used in the original PROCESS Model 8 analyses. Simulation-based methods can accommodate conditional indirect effects involving categorical predictors and moderators (Donnelly et al., 2023). For the simulations, social comparison was coded X = 0 for upward and X = 1 for downward comparison; regulatory focus was coded W = 0 for promotion and W = 1 for prevention focus. This 0/1 coding is a recoding of the 1/2 coding used in the original PROCESS analyses and does not change the interaction coefficient or the index of moderated mediation. The mediator M was the self-evaluation threat composite. We evaluated preference-adjusted and classical SoMi proportions as separate outcomes Y. No covariates were included. The model equations were:

M = a0 + a1X + a2W + a3XW + eM

Y = c0 + c1X + c2W + c3XW + bM + eY

The conditional indirect effect was calculated as $(a_1 + a_3W)b$, and the index of moderated mediation was a_3b. The fitted coefficients in Table S2 provided the reference values for the simulation scenarios. Each simulated dataset retained the observed cell sizes: 36 participants in the promotion/upward condition, 36 in the promotion/downward condition, 35 in the prevention/upward condition, and 37 in the prevention/downward condition. Mediator and outcome values were generated sequentially from the specified equations using independent normally distributed residuals with the standard deviations reported in Table S2. Both equations were then fitted using ordinary least squares. Analyses were conducted in R 4.6.0.

Table S2. Reference regression coefficients used to calibrate scenarios.

Equation	Intercept	X	W	XW	M	Residual SD
Self-evaluation threat	2.82407	−.37037	.69974	−.80209	—	.82025
Preference-adjusted SoMi	.25115	.03150	−.05440	.09081	−.02746	.13901
Classical SoMi	.70113	.04128	−.10641	.16761	−.05729	.22737

Simulation results

Table S3. Estimated power for the index of moderated mediation at N = 144.

Path multiplier (k)	Preference-adjusted SoMi: assumed index	Estimated power	Classical SoMi: assumed index	Estimated power
0.50	.00551	.032	.01149	.062
0.75	.01239	.141	.02585	.237
1.00	.02202	.389	.04595	.544

1.25	.03441	.629	.07179	.810
1.50	.04955	.792	.10338	.951
2.00	.08809	.966	.18380	.992

Note. Simulations followed the PROCESS Model 8 structure and retained the observed allocation across the four experimental conditions. The multiplier k was applied to both the social comparison × regulatory focus coefficient in the mediator equation (a_3) and the mediator coefficient in the outcome equation (b); consequently, the assumed index (a_3b) changed by k^2. Remaining coefficients and residual standard deviations were held constant. Each scenario included 2,000 simulated datasets. Estimated power was the proportion of datasets in which a 95% Monte Carlo confidence interval, constructed from the products of 5,000 independent normal draws based on the estimated a_3 and b coefficients and their standard errors, excluded zero. These estimates are conditional on the specified simulation assumptions and use a different interval procedure from the nonparametric percentile bootstrap used in the original analyses.

Simulation-based power estimates for both SoMi outcomes are presented in Table S3. Estimated power increased with the magnitudes of the component paths. In the reference scenarios (k = 1.00), power was .389 for preference-adjusted SoMi and .544 for classical SoMi. For preference-adjusted SoMi, power approached .80 when both paths were multiplied by 1.50 (.792). For classical SoMi, power exceeded .80 when both paths were multiplied by 1.25 (.810).

These analyses examined sensitivity to the specified path-effect scenarios. The simulations treated SoMi scores as continuous outcomes and assumed independent normally distributed residuals. They used Monte Carlo confidence intervals, whereas the original analyses used nonparametric percentile-bootstrap intervals. The power estimates therefore pertain to the simulation assumptions and interval procedure.

Reference: Donnelly, S., Jorgensen, T. D., & Rudolph, C. W. (2023). Power analysis for conditional indirect effects: A tutorial for conducting Monte Carlo simulations with categorical exogenous variables. *Behavior Research Methods, 55*, 3892–3909. https://doi.org/10.3758/s13428-022-01996-0

Supplementary Material S9: Distributional Diagnostics and Robustness Analyses

Preference-adjusted SoMi scores contained 35.9%, 28.0%, and 14.6% zero values in Experiments 1–3, respectively. ANOVA residuals departed from normality (Shapiro–Wilk *p*s < .001); median-centred Levene tests yielded p = .370, .069, and .059. The Experiment 3 ANOVA additionally showed evidence of heteroscedasticity (Breusch–Pagan p = .030). Robustness analyses used participant-level quasi-binomial models with a logit link and estimated dispersion, modelling the number of matched trial pairs meeting the preference-adjustment criterion out of 12.

Table S4. Quasi-binomial robustness tests for preference-adjusted SoMi.

Experiment	Effect	*F*	*df*	*p*
1	Comparison condition	3.50	2, 164	.032
2	Comparison condition	2.77	2, 161	.066
3	Comparison × regulatory focus	6.52	1, 140	.012

Note. Overall effects were evaluated using quasi-binomial deviance F tests.

In Experiment 2, the self-evaluation threat coefficient remained negative and statistically significant with HC3 standard errors and quasi-binomial regression (both ps < .001). In Experiment 3, the corresponding coefficient was also negative, with p = .043 and .031, respectively. These tests evaluate the individual outcome-regression coefficient rather than the index of moderated mediation.
